# Modular evolution of secretion systems and virulence plasmids in a bacterial species complex

**DOI:** 10.1186/s12915-021-01221-y

**Published:** 2022-01-13

**Authors:** Lin Chou, Yu-Chen Lin, Mindia Haryono, Mary Nia M. Santos, Shu-Ting Cho, Alexandra J. Weisberg, Chih-Feng Wu, Jeff H. Chang, Erh-Min Lai, Chih-Horng Kuo

**Affiliations:** 1 Institute of Plant and Microbial Biology, Academia Sinica, Taipei, Taiwan; 2 Molecular and Biological Agricultural Sciences Program, Taiwan International Graduate Program, National Chung Hsing University and Academia Sinica, Taipei, Taiwan; 3 Graduate Institute of Biotechnology, National Chung Hsing University, Taichung, Taiwan; 4 Department of Botany and Plant Pathology, Oregon State University, Corvallis, Oregon, USA; 5 Biotechnology Center, National Chung Hsing University, Taichung, Taiwan

**Keywords:** *Agrobacterium*, Genome, Secretion system, Virulence, Plasmid, Molecular evolution

## Abstract

**Background:**

Many named species as defined in current bacterial taxonomy correspond to species complexes. Uncertainties regarding the organization of their genetic diversity challenge research efforts. We utilized the *Agrobacterium tumefaciens* species complex (a.k.a. *Agrobacterium* biovar 1), a taxon known for its phytopathogenicity and applications in transformation, as a study system and devised strategies for investigating genome diversity and evolution of species complexes.

**Results:**

We utilized 35 genome assemblies, including 14 newly generated ones, to achieve a phylogenetically balanced sampling of *A. tumefaciens*. Our genomic analysis suggested that the 10 genomospecies described previously are distinct biological species and supported a quantitative guideline for species delineation. Furthermore, our inference of gene content and core-genome phylogeny allowed for investigations of genes critical in fitness and ecology. For the type VI secretion system (T6SS) involved in interbacterial competition and thought to be conserved, we detected multiple losses and one horizontal gene transfer. For the tumor-inducing plasmids (pTi) and pTi-encoded type IV secretion system (T4SS) that are essential for agrobacterial phytopathogenicity, we uncovered novel diversity and hypothesized their involvement in shaping this species complex. Intriguingly, for both T6SS and T4SS, genes encoding structural components are highly conserved, whereas extensive diversity exists for genes encoding effectors and other proteins.

**Conclusions:**

We demonstrate that the combination of a phylogeny-guided sampling scheme and an emphasis on high-quality assemblies provides a cost-effective approach for robust analysis in evolutionary genomics. We show that the T6SS VgrG proteins involved in specific effector binding and delivery can be classified into distinct types based on domain organization. The co-occurrence patterns of VgrG-associated domains and the neighboring genes that encode different chaperones/effectors can be used to infer possible interacting partners. Similarly, the associations between plant host preference and the pTi type among these strains can be used to infer phenotype-genotype correspondence. Our strategies for multi-level investigations at scales that range from whole genomes to intragenic domains and phylogenetic depths from between- to within-species are applicable to other bacteria. Furthermore, modularity observed in the molecular evolution of genes and domains is useful for inferring functional constraints and informing experimental works.

**Supplementary Information:**

The online version contains supplementary material available at 10.1186/s12915-021-01221-y.

## Background

Understanding bacterial biology, notably for purposes of tackling pathogenicity, requires the ability to identify biological entities [1, 2] at the species level and to infer their evolutionary relationships. However, many bacterial groups are currently unresolved and are classified as species complexes. These uncertainties regarding species boundaries hamper research, communication, and policy-making such as in healthcare guidelines, pathogen quarantine regulations, and biological resource management. Based on barriers to homologous recombination, an analysis of > 20,000 bacterial genome sequences from 91 species belonging to 13 phyla found that 21 of the previously recognized species comprise multiple biological species [3]. These 21 groups include those that are important as pathogens (e.g., *Mycobacterium tuberculosis*, *Pseudomonas aeruginosa*, and *Vibrio cholerae*) or beneficial microbes (e.g., *Lactobacillus casei* and *Sinorhizobium meliloti*). This finding highlights the ubiquity of species complexes across bacterial lineages, even for those that are extensively studied.

For such complexes, comprehensive understanding of the genetic diversity organization is required for robust species delineation, which in turn is essential for providing a reliable framework to interpret experimental findings and to gain insights into the biology. The use of genomic information has long been suggested as a powerful approach for defining species boundaries because the comprehensive genetic information can provide definitive and potentially quantitative guidelines [4]. However, several issues regarding genomic studies of bacterial species have remained unresolved. First, while genomospecies defined by overall genome divergence were suggested to represent distinct biological entities [5–9], the exact criteria for establishing the species boundaries are disputed. Although 95% average nucleotide identity (ANI) across the conserved parts of genomes was proposed as a universal boundary for defining species in bacteria [10], this criterion was challenged [11]. Additionally, ANI values alone do not provide information such as gene content or phylogenetic relationships, which are critical in understanding biological entities [4]. Second, phylogenetic relationships among closely related bacterial strains often cannot be resolved with confidence, yet such information is fundamental for evolutionary analysis. Third, comparative genomics studies are often limited by taxon sampling and/or assembly quality of available genome sequences.

In this study, we utilized the *Agrobacterium tumefaciens* species complex, also known as *Agrobacterium* biovar 1 [12], as the study system for developing strategies that provide appropriate sampling and utilize multifaceted genomic analysis to resolve species boundaries and to investigate molecular evolution of key traits. These bacteria are known as the causative agents of crown gall disease that affects over 90 plant families [13]. More importantly, the development of *Agrobacterium*-mediated transformation has provided a critical tool for genetic manipulation in plant sciences and agricultural biotechnology [14, 15]. Due to their importance, this complex has been studied for over a century and was found to harbor extensive phenotypic and genetic diversity that continues to confound efforts to resolve their taxonomy [12–14]. Various methods, such as DNA-DNA hybridization, biochemical characteristics, and molecular markers have been used to define 10 genomospecies (i.e., G1-G9 and G13), which have continually been associated to new nomenclature, a process that causes greater confusion than resolution [5, 6, 16–18]. For example, the reference strain C58 used in many *A. tumefaciens* studies [15, 19, 20] belongs to G8, for which the name *Agrobacterium fabrum* was proposed in 2011 [21]. This has resulted in mixed usage of two names with different meanings (i.e., *A. tumefaciens* for the entire complex and *A. fabrum* for G8) in databases and literature. Compounding confusion, the name *Agrobacterium radiobacter* refers to *A. tumefaciens* G4 [18, 21] and is also a heterotypic synonym of *A. tumefaciens* [22]. Hereafter, we use *A. tumefaciens* in reference to the entire species complex and specific designations (i.e., G1–G9 and G13) for the genomospecies.

Previous characterizations found that *A. tumefaciens* strains have multipartite genomes with one circular chromosome, one linear chromosome, and highly variable plasmids [9, 23–25]. Consistent with the high levels of genetic divergence inferred from DNA-DNA hybridization [5], cross-genomospecies comparisons typically found that < 80% of the genes are conserved [7, 26]. Strikingly, > 32,000 horizontal gene transfer (HGT) events have been inferred to have shaped the evolutionary history of *A. tumefaciens* [8]. Because the HGT patterns indicated co-transfers of genes that encode coherent biochemical pathways, it was hypothesized that purifying selection on those acquired gene clusters and overall gene content drove the ecological diversification among genomospecies [8]. Moreover, the oncogenic plasmids that determine *Agrobacterium* phytopathogenicity exhibit complex modularity and transmission patterns, which further contributed to the diversification of these pathogens and their global spread [9]. However, despite the progresses, those better-characterized genomospecies (e.g., G1, G4, G7, and G8) and pathogenic strains were highly overrepresented in previous genomics studies [7–9], and such biases may affect our understanding of agrobacterial diversity and evolution.

To develop effective strategies for investigating bacterial species complexes such as *A. tumefaciens*, we started by performing targeted genome sequencing for strains in underrepresented lineages to achieve a balanced taxon sampling of the study system. The sampling scheme was based on information from two previous phylogenetic analyses of the *A. tumefaciens* species complex and its sister lineages, one based on *recA* [18] and the other based on 24 conserved genes [9]. We also limited analyses to only high-quality assemblies, which enabled detailed examinations of replicon-level synteny and confident inferences of gene presence/absence. The global view of genomic diversity and resolved phylogeny provided a robust framework for focused investigations of the genetic elements involved in key aspects of agrobacterial fitness and ecology, namely the type VI secretion system (T6SS) for interbacterial competition [7, 27] and the virulence plasmids for phytopathogenicity [13–15]. Taken together, the investigations, scaling from whole-genome, whole-replicon, gene clusters, individual genes, and intragenic protein domains, provided novel and detailed information on the evolution and genetic diversity of bacteria important in plant pathology and biotechnology. Moreover, the strategies developed in this work are applicable to the study of other bacterial species complexes.

## Results

### Genome sampling, molecular phylogeny, and divergence

Based on existing knowledge of *A. tumefaciens* diversity [5, 6, 16–18] and availability of genomic resources [7–9], we identified 12 strains that represent six poorly characterized genomospecies (Table 1). Additionally, two *Agrobacterium larrymoorei* strains, which represent the most closely-related sister lineage of *A. tumefaciens* [9, 18], were included as the outgroup. Whole-genome sequencing with substantial efforts in iterative improvements of the assemblies based on experimental and bioinformatic approaches were conducted for these 14 strains. Additionally, we selected 21 representatives from the 98 *A. tumefaciens* genome assemblies available from GenBank [28] (Table 1) to yield a dataset with maximal genetic diversity without emphasis on including pathogenic strains. To ensure balanced sampling, we selected between two and five strains for each of the 10 recognized *A. tumefaciens* genomospecies. Importantly, 19 of these 35 assemblies, including nine produced in this study, are complete and most others are nearly complete (i.e., average N50 = 1.3 Mb; cf. the two chromosomes are ~ 2.9 and ~ 2.3 Mb, respectively).

**Table 1:** List of the genome sequences used in this study. These include 14 new genomes derived from this study and 21 additional representatives from GenBank. Two *Agrobacterium larrymoorei* strains are included as the outgroup. Species name abbreviations: *At*, *Agrobacterium tumefaciens*; *Al*, *Agrobacterium larrymoorei*

Species	Strain	Accession	Assembly	Coding sequences	Pseudo-genes	Geographic origin	Isolation source
*At* G1	1D1108	GCF_003666425	Complete	5312	167	MD, USA	*Euonymus* sp.
*At* G1	5A	GCF_000236125	50 contigs	5343	169	MT, USA	Soil
*At* G1	Ach5	GCF_000971565	Complete	5184	145	CA, USA	*Achillea ptarmica*
*At* G1	N2/73	GCF_001692195	29 scaffolds	5345	157	OR, USA	Cranberry
*At* G1	S56	GCF_900014385	6 scaffolds	5414	218	?	Plant
*At* G2	CFBP5494	GCF_900013495	5 scaffolds	5469	233	France	Human
*At* G2	CFBP5496	GCF_005144405	9 contigs	5137	223	France	Human
*At* G2	CFBP5875	GCF_005221365	Complete	4570	146	Belgium	Ditch water
*At* G3	CFBP6623	GCF_005221385	Complete	5081	180	France	Antiseptic flask
*At* G3	CFBP6624	GCF_005221425	Complete	5157	148	France	Human
*At* G4	183	GCF_004023565	Complete	5051	199	Tunisia	*Prunus dulcis*
*At* G4	186	GCF_002591665	Complete	5298	207	CA, USA	*Juglans regia*
*At* G4	12D1	GCF_003667905	Complete	5005	167	?	?
*At* G4	1D1460	GCF_003666445	Complete	5290	269	CA, USA	*Rubus* sp.
*At* G5	CFBP6625	GCF_005221465	Complete	5446	198	France	Food
*At* G5	CFBP6626	GCF_005221445	Complete	5080	199	France	Human
*At* G5	F2	GCF_000219665	8 contigs	5085	135	Harbin, China	Soil
*At* G6	CFBP5499	GCF_005221325	Complete	5654	240	South Africa	*Dahlia* sp.
*At* G6	CFBP5877	GCF_005221345	Complete	5463	236	Israel	*Dahlia* sp.
*At* G7	1D1609	GCF_002943835	Complete	5539	254	CA, USA	*Medicago sativa*
*At* G7	CFBP4996	GCF_005144435	10 contigs	5741	213	UK	*Flacourtia ramontchi*
*At* G7	CFBP7129	GCF_005221405	Complete	5927	285	Tunisia	*Pyrus communis*
*At* G8	12D13	GCF_003667945	Complete	5083	178	?	?
*At* G8	1D132	GCF_003667725	Complete	5176	169	CA, USA	*Cerasus pseudocerasus*
*At* G8	ATCC31749	GCF_002916755	4 contigs	4560	811	China	Plant
*At* G8	C58	GCF_000092025	Complete	5355	29	NY, USA	*Cerasus pseudocerasus*
*At* G9	CFBP5506	GCF_005144495	15 contigs	4236	157	Australia	Soil
*At* G9	CFBP5507	GCF_005144505	16 contigs	5299	253	Australia	Soil
*At* G9	GBBC3283	GCF_007002765	48 contigs	4946	203	Belgium	*Solanum lycopersicum*
*At* G13	CFBP6927	GCF_900012615	9 scaffolds	4722	94	France	Rhizospheric soil from *Prunus persicae*
*At* G13	S2	GCF_000723345	60 contigs	5574	167	?	?
*At* G21	MAFF210266	GCF_007002865	26 contigs	5199	162	Japan	*Cucumis melo*
*At* G22	KCJK1736	GCF_001641425	41 contigs	4933	162	FL, USA	*Bos taurus* feces
*Al*	CFBP5473	GCF_005145045	Complete	4777	142	FL, USA	*Ficus benjamina*
*Al*	CFBP5477	GCF_005144425	24 contigs	4657	95	Italy	?

**Fig. 1:**
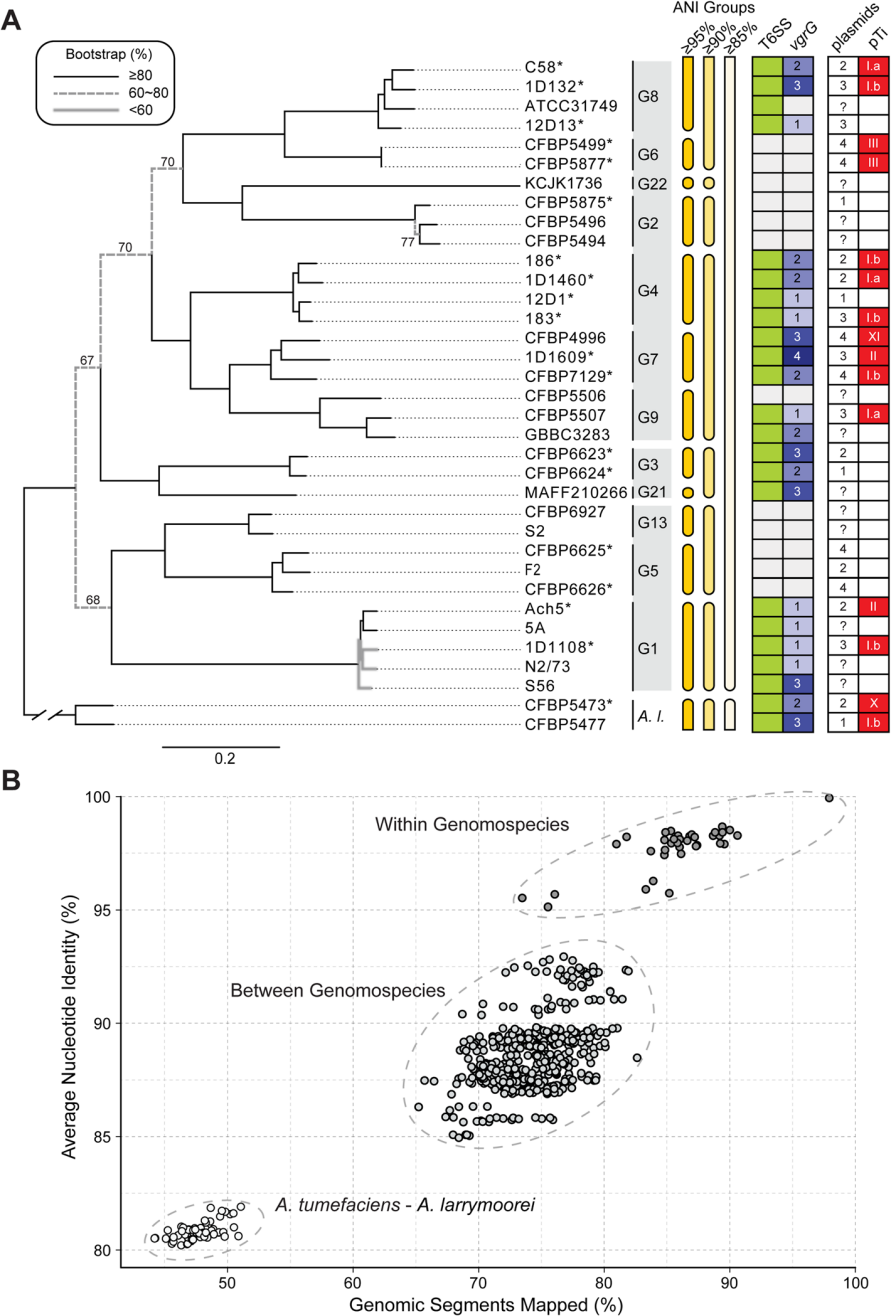
Relationships among representatives of the *Agrobacterium tumefaciens* species complex. The sister species *Agrobacterium larrymoorei* (*A. l.*) is included as the outgroup. **A** Maximum likelihood phylogeny based on a concatenated alignment of 2093 single-copy genes shared by all 35 genomes (635,594 aligned amino acid sites). Bootstrap support values in the range of 60–80% are labeled. Strains with complete genome assemblies are highlighted with an asterisk (“*”). The genomospecies assignments (i.e., G1-G9, G13, and G21-G22) are labeled to the right of strain names. The three *A. tumefaciens* supergroups are indicated by the colored background of the genomospecies assignments. Information to the right of the genomospecies assignments shows the grouping of genomes according to different cutoff values of genome-wide average nucleotide identity (ANI), the presence/absence of type VI secretion system (T6SS)-encoding gene cluster (green: present; white: absent), copy number of *vgrG* (white background: absent), number of plasmids, and the tumor-inducing plasmid (pTi) type based on k-mer profile (white background: absent). **B** Pairwise genome similarities based on the percentages of genomic segments mapped and the ANI values

Based on the homologous gene clustering results among these strains, we identified a core genome of 2093 single-copy genes, which correspond to ~ 40% of the genes annotated in each individual genome sequence. Compared to previous studies that conducted genome-based phylogenetic analysis for *Agrobacterium* or higher taxonomic ranks [8, 9, 29], the more focused sampling in this study yielded a higher core gene count by one-to-two orders of magnitude. This increase in core gene count and the improvement in taxon sampling allowed for the inference of a well-resolved maximum likelihood phylogeny of the *A. tumefaciens* species complex (Fig. 1A). Each of the 10 currently recognized genomospecies forms a distinct monophyletic clade with > 80% bootstrap support. Additionally, we identified two novel genomospecies, G21 and G22, each represented by a single strain. The pattern of overall genome similarities exhibits a discrete multimodal distribution that supports use of a 95% ANI cutoff for delineating bacterial species [10] and quantifies the divergence of the *A. tumefaciens* complex from its most closely related sister lineage (Fig. 1B).

These 12 *A. tumefaciens* genomospecies were classified into seven groups based on 90% ANI and further assigned to three supergroups according to the phylogeny (Fig. 1A). Based on the time-calibrated phylogeny reported in Weisberg et al. [9], the most recent common ancestor (MRCA) of *A. tumefaciens* emerged ~ 48 million years ago (Mya) with a 95% highest posterior density (HPD) interval of 38.5–58.0 Mya, the three supergroups diverged ~ 40 Mya (95% HPD interval 31.5–48.1 Mya), and most of the recognized genomospecies emerged ~ 2–7 Mya (95% HPD interval 1.2–9.7 Mya). The large 95% HPD intervals suggest uncertainties regarding these time estimates. Regardless of the exact divergence time, the inferred rapid radiation in the early history of *A. tumefaciens* as shown by the short branch lengths likely prevented confident resolution of those deeper relationships in previous studies [8, 9, 29]. With improvements in the taxon sampling of this work, we observed ~ 70% bootstrap support for those early nodes (Fig. 1A). This organismal tree provides a strong framework for our downstream examination of gene and domain phylogenies.

The high levels of assembly completeness provided confident inference of gene content comparisons. The principal coordinate analysis and hierarchical clustering results indicated that all 12 *A. tumefaciens* genomospecies are similar to one another while distinct from *A. larrymoorei* (Additional file 1: Figure S1A and S1C). Nonetheless, with the exception of G4 and G7, these genomospecies are distinguishable based on gene content (Additional file 1: Figure S1B and S1D). This finding suggests that despite the extensive HGT inferred within this complex [8], the genomospecies defined by 95% ANI likely represent distinct biological entities.

### Diversity and evolution of the T6SS genes

The confident inference of organismal phylogeny and gene content afforded by high-quality genome assemblies provided a robust framework for evolutionary analysis of key traits, particularly for those influence by the absence or loss of genes. We first focus on genes encoding the T6SS, a phage tail-like contractile nanomachine commonly found in Proteobacteria and used to inject effectors into eukaryotic or bacterial cells. The T6SS has major roles in pathogenesis, symbiosis, and interbacterial competition [30–33]. For *A. tumefaciens*, the T6SS is a key weapon for in planta competition between different genomospecies [7] and against other bacteria [27]. Thus, investigating the diversity and evolution of T6SS genes may shed light on a trait that influences the ecology and evolution of *A. tumefaciens*.

**Fig. 2:**
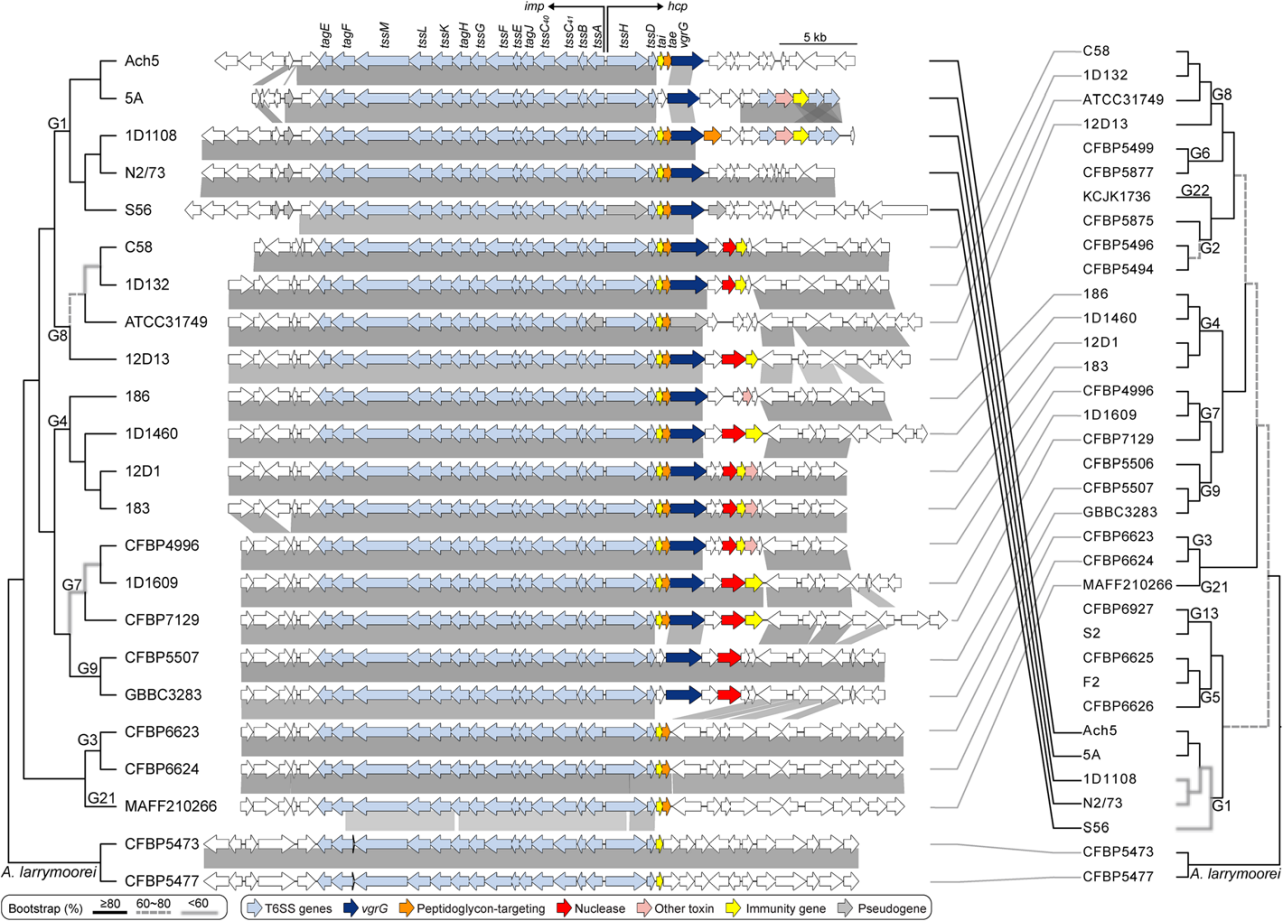
Phylogeny and organization of the T6SS gene cluster. The maximum likelihood phylogeny was inferred based on a concatenated alignment (5960 aligned amino acid sites) of 14 core T6SS genes, including *tagE*, *tagF*, *tssM*, *tssL*, *tssK*, *tagH*, *tssG*, *tssF*, *tssE*, *tagJ*, *tssC40*, *tssC41*, *tssB*, and *tssD*. Two other core genes, *tssA* and *tssH*, are excluded because some homologs are pseudogenized. Genes downstream of *tssD* (e.g., *tai*, *tae*, and *vgrG*) are excluded due to variable presence. The species phylogeny on the right is based on Fig. 1. Genes are color-coded according to annotation, and syntenic regions are indicated by gray blocks

In a previous study that examined four *A. tumefaciens* genomospecies, T6SS-mediated anti-bacterial activity was observed for all 11 strains sampled and thought to be a conserved trait of this species complex [7]. To our surprise, among the 33 *A. tumefaciens* strains examined in this work, a patchy distribution of the T6SS genes was observed (Fig. 1). Gene absences are in strains corresponding to previously under-characterized genomospecies and were confirmed by examining syntenic regions and using TBLASTN [34] to search entire genome sequences. For strains encoding a T6SS, corresponding genes are consistently located on the linear chromosome and mostly form a cluster of ~ 20 genes organized as two adjacent and oppositely oriented *imp* and *hcp* operons [7, 35] (Fig. 2). Some strains harbor accessory loci containing *vgrG* (involved in effector delivery) and other T6SS genes located elsewhere on the linear chromosome [7, 33, 35] (Additional file 1: Figure S2). The T6SS gene phylogeny is largely congruent with the species tree (Fig. 2). One notable exception is that the MRCA of G1 appears to have acquired the T6SS genes from a G8-related donor. Consistent with this inference, the T6SS genes in G1 strains are located in a different chromosomal location compared to strains of other genomospecies (Additional file 1: Figure S2). Based on these observations, it is likely that a T6SS gene cluster was present in the MRCA of G8-G6-G14-G2-G4-G7-G9-G3-G15 and at least two independent losses have occurred in G6 and G14-G2. Regarding the ancestral state in the MRCA of the *A. tumefaciens* complex, presence of the T6SS genes appears to be a more parsimonious hypothesis based on the presence of these genes in the outgroup (Fig. 2). However, the lack of synteny conservation between the linear chromosomes of *A. tumefaciens* and *A. larrymoorei* and the variable locations of *vgrG* homologs (Additional file 1: Figure S2) suggest that multiple independent origins are also possible. For broader scales, the T6SS genes have a patchy distribution among Rhizobiaceae [33, 36], indicating that these genes are not essential for these bacteria and have high rates of gains and losses. Consistent with this, multiple pseudogenes confirmed by manual curation of annotation were found (e.g., *tssH* in S56 and *tssA*/*vgrG* in ATCC31749) (Fig. 2), suggesting that for some strains these gene clusters are in the process of degradation and will be eventually lost.

Examination of synteny revealed that the *imp* operon, which encodes the majority of T6SS structural components [37], is more conserved in gene composition and order than the *hcp* operon, which often has different genes downstream of *vgrG* (Fig. 2). This genetic diversity may play a key role in interbacterial competition because genes downstream of *vgrG* include those that encode effector and immunity (EI) protein pairs [7]. The agrobacterial T6SS effectors often correspond to different toxins, and the cognate immunity proteins provide protection against self-intoxication [7, 27, 33, 36]. The rapid evolution of EI gene pairs is illustrated by three examples. First, despite the low levels of sequence divergence among G1 strains (i.e., > 98% ANI in all pairwise comparisons), different genes are found downstream of their *vgrG* homologs and this variation is not consistent with either the species phylogeny or the T6SS core gene phylogeny. Second, strain CFBP4996 of G7 has homologs of the same EI gene pair as strains 12D1 and 183 of G4, rather than with other members of G7. Third, in both G3 strains, *vgrG* and the associated EI genes are located elsewhere on the linear chromosome, rather than being a part of the *hcp* operon (Fig. 2 and Additional file 1: Figure S2). These results suggest that recombination involving gene modules has contributed to the genetic diversity of these *A. tumefaciens* T6SS EI gene pairs.

### Modularity of VgrG and its associated EI pair

**Fig. 3:**
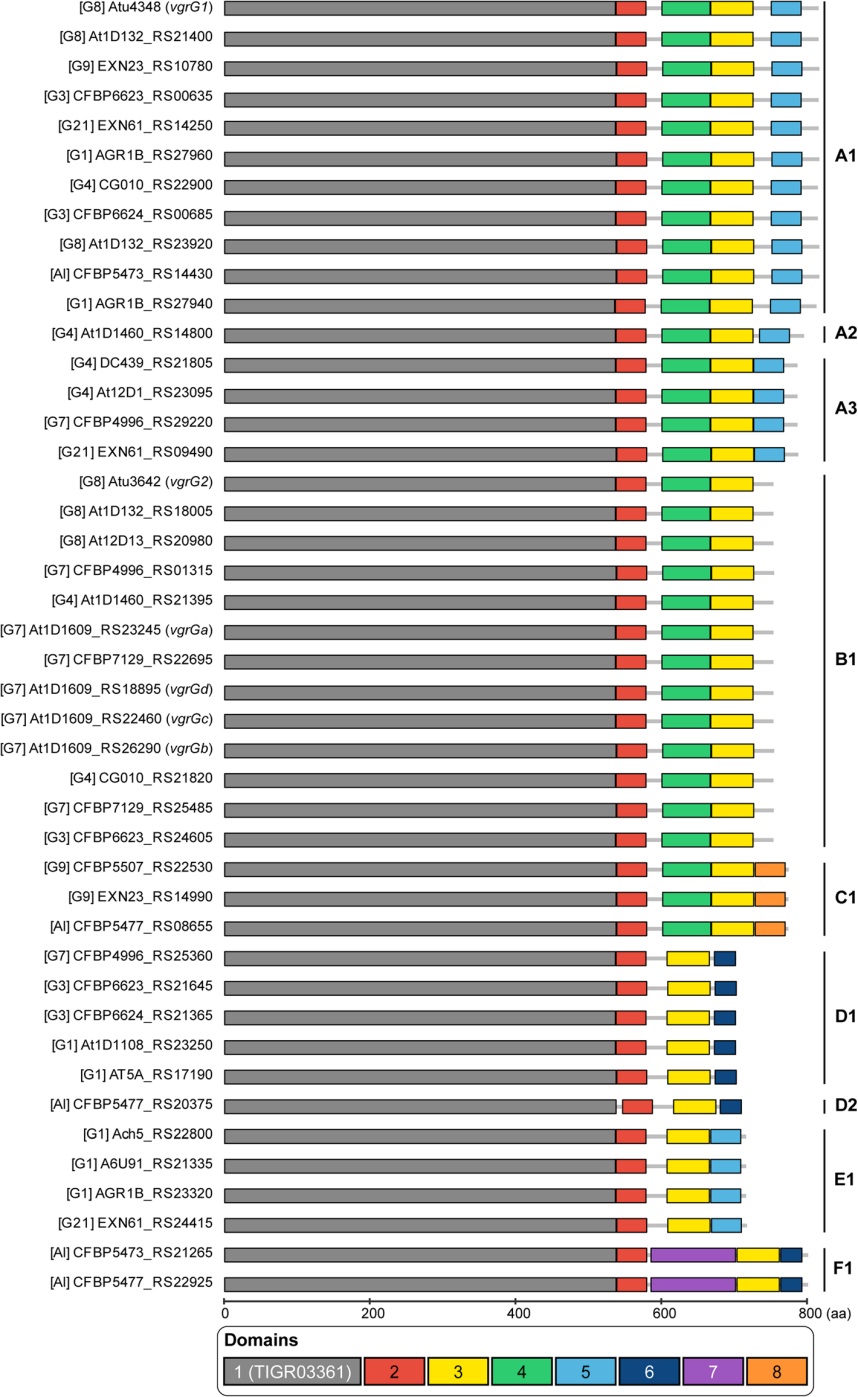
Domain organization and classification of *vgrG* homologs. Six major types with two types having subtypes (A1-3 and D1-2) within them were identified and labeled on the right. For each homolog, the genomospecies assignment is provided in square brackets, followed by the locus tag. The gene names are provided in parenthesis for those functionally characterized homologs (i.e., *vgrG1-2* for C58 homologs and *vgrGa-d* for 1D1609 homologs)

**Fig. 4:**
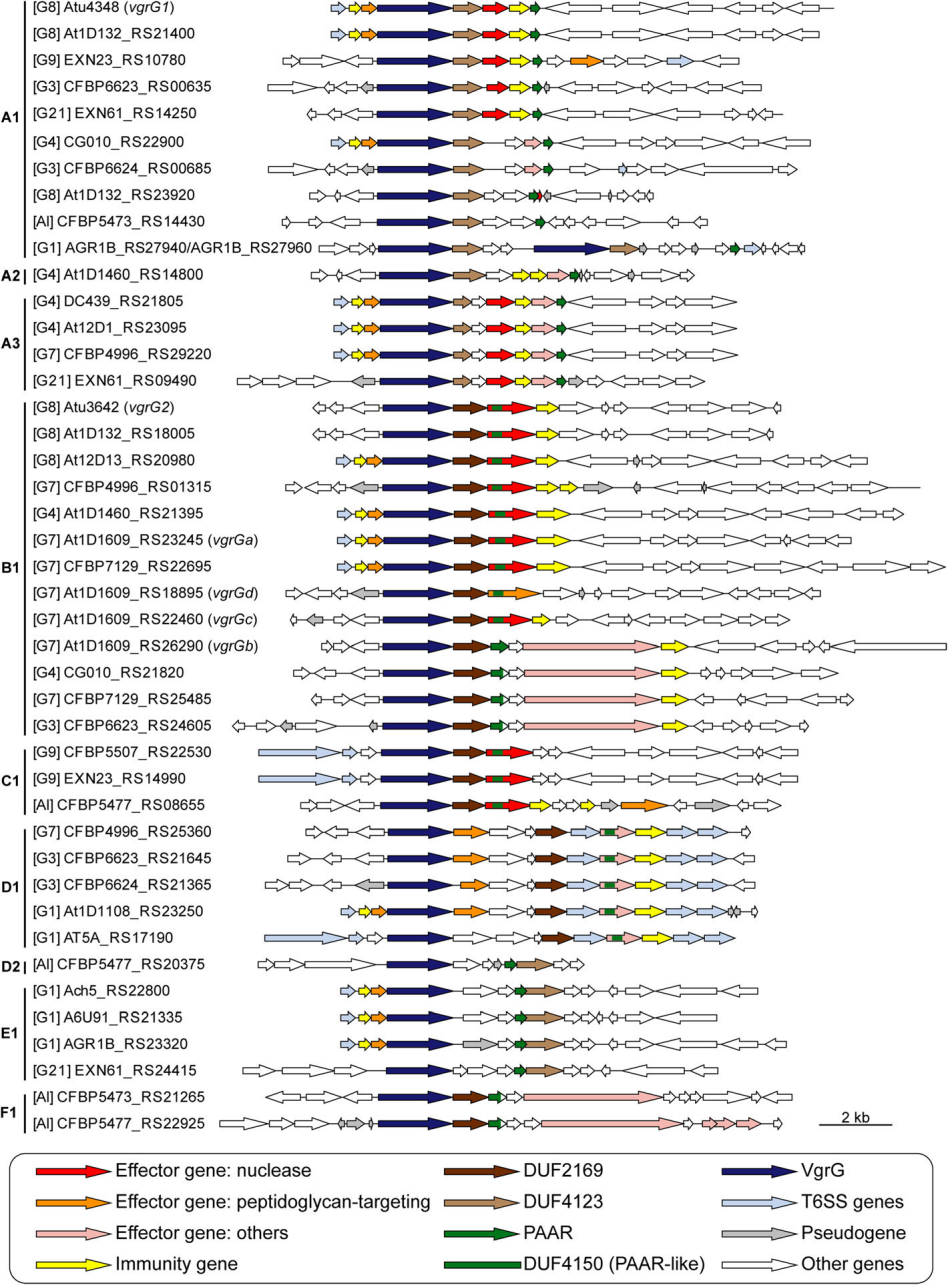
Gene neighborhoods of *vgrG* homologs. The grouping and labeling of *vgrG* homologs are based on the convention used in Fig. 3. For each *vgrG* homolog, three upstream genes are plotted to illustrate if it is associated with the main T6SS gene cluster or not, and 10 downstream genes are plotted to illustrate putative effector/immunity genes. Two A1-type homologs from the strain S56 (i.e., AGR1B_RS27940 and AGR1B_RS27960) are in close association with each other and plotted together

The knowledge that *vgrG* homologs encode proteins with distinct C-terminal domains responsible for binding specificities of different T6SS effectors for delivery suggested that each *vgrG* homolog and its downstream EI gene pair may evolve as a functional module [33, 36]. Here, we sought to investigate the patterns of gene co-occurrence and intra-module recombination to better understand the diversity and evolution of these genes. For in-depth investigation of *vgrG* evolution, we began by examining domain architecture of VgrG proteins and uncovered eight distinct domains (Additional file 1: Figure S3). Based on differences in domain composition, the 44 *vgrG* homologs, including 17 associated with the main T6SS gene cluster and 27 associated with accessory loci, were classified into six major types and nine subtypes (Fig. 3). Only three of the domains are present in all VgrG variants. The N-terminal domain 1 is the most conserved (Additional file 1: Figure S3) and the only one found in databases. This domain corresponds to TIGR03361, which accounts for ~ 66–77% of the protein length and the bulk of the structures that forms a trimeric complex analogous to a phage tail spike [38, 39] (Additional file 1: Figure S4). It is worth noting that the C-terminal end of domain 1 was identified as a recombination hotspot in a related study on agrobacterial T6SS genes [40]. For domain 5 that was found in all *vgrG* homologs belonging to subtypes A1-A3 and E1 (Fig. 3), the presence of this domain is perfectly correlated with the presence of a downstream DUF4123-domain-containing gene (Fig. 4). Because this DUF4123 domain acts as an adaptor/chaperone for effector loading onto VgrG in *A. tumefaciens* [36] and *Vibrio cholerae* [41, 42], this strong co-occurrence suggests specific interactions between VgrG domain 5 and DUF4123. Thus, combining domain analysis with gene co-occurrence provides a new strategy for predicting the interacting domains of VgrG with other T6SS components.

Intriguingly, despite conservation of domain architecture within each subtype (Fig. 3), the phylogenies inferred from the three domains encoded by all *vgrG* homologs do not have the same topology (Additional file 1: Figure S5). For domain 1, sequences from the same subtype do not always form monophyletic groups. For domains 2 and 3, the short sequence lengths limited the phylogenetic resolution; nonetheless, low divergence within the same subtype and high divergence between subtypes were observed. These patterns suggest that each domain-encoding region evolved independently and can recombine between subtypes.

At the level of gene cluster organization, *vgrG* homologs within a subtype can have distinct downstream genes (e.g., A1 and B1), regardless of whether they are associated with the main T6SS gene cluster (Fig. 4 and Additional file 2: Dataset S1). These findings suggest that in addition to domain shuffling among *vgrG* homologs [40], recombination also facilitated novel *vgrG*-effector pairings in the evolution of these T6SS genes.

**Fig. 5:**
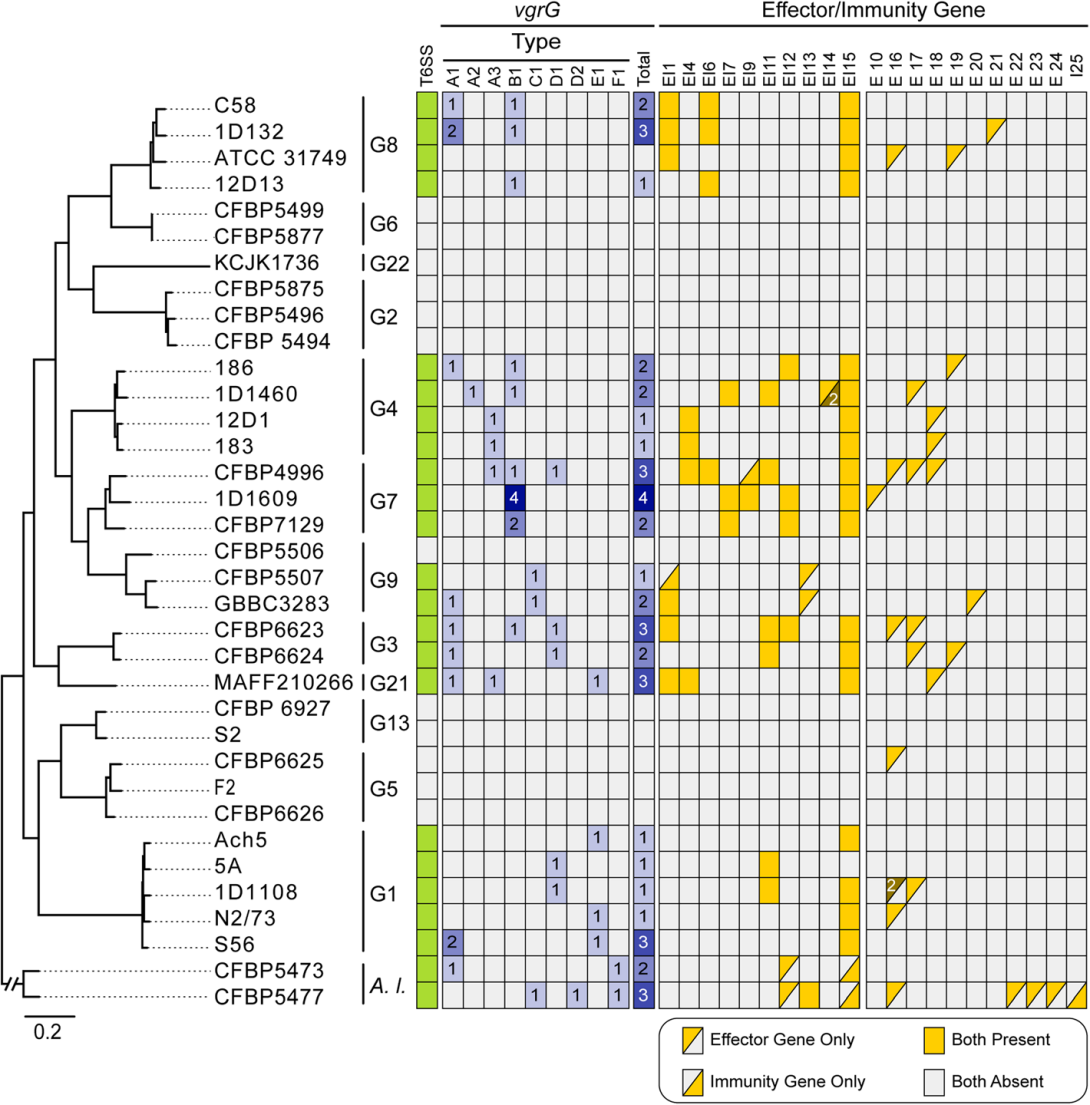
Phylogenetic distribution of *vgrG* homologs and T6SS effector/immunity genes. The species tree is based on Fig. 1. Gene presence is illustrated with colored cells in the heatmap, gene copy numbers are labeled when applicable

When T6SS diversity was examined in a phylogenetic context, numbers, and types of *vgrG* homologs, as well as their linked EI genes, lack strong correlations with species phylogeny (Fig. 5). Based on our manual curation of *vgrG*-associated genes, a total of 63 putative effector genes were identified (Additional file 2: Dataset S2). Among these, peptidoglycan-targeting toxins and nucleases are the two most commonly found categories with 21 each. This finding is consistent with an investigation of > 1000 T6SS effectors sampled from 466 species in the phylum Proteobacteria [32], which also found these two as the dominant categories and suggested that they play complementary roles in T6SS-mediated competition. Based on our homologous gene clustering results, these 63 putative effector genes were classified into 20 families (Fig. 5). Among these, 11 were experimentally validated in previous studies, including EI1/6/15 in Ma et al. [27], EI4/11 in Wu et al. [7], EI7 in Santos et al. [33], and EI2/5/8/9/10 in Wu et al. [40].

### The virulence plasmids and associated genes

**Fig. 6:**
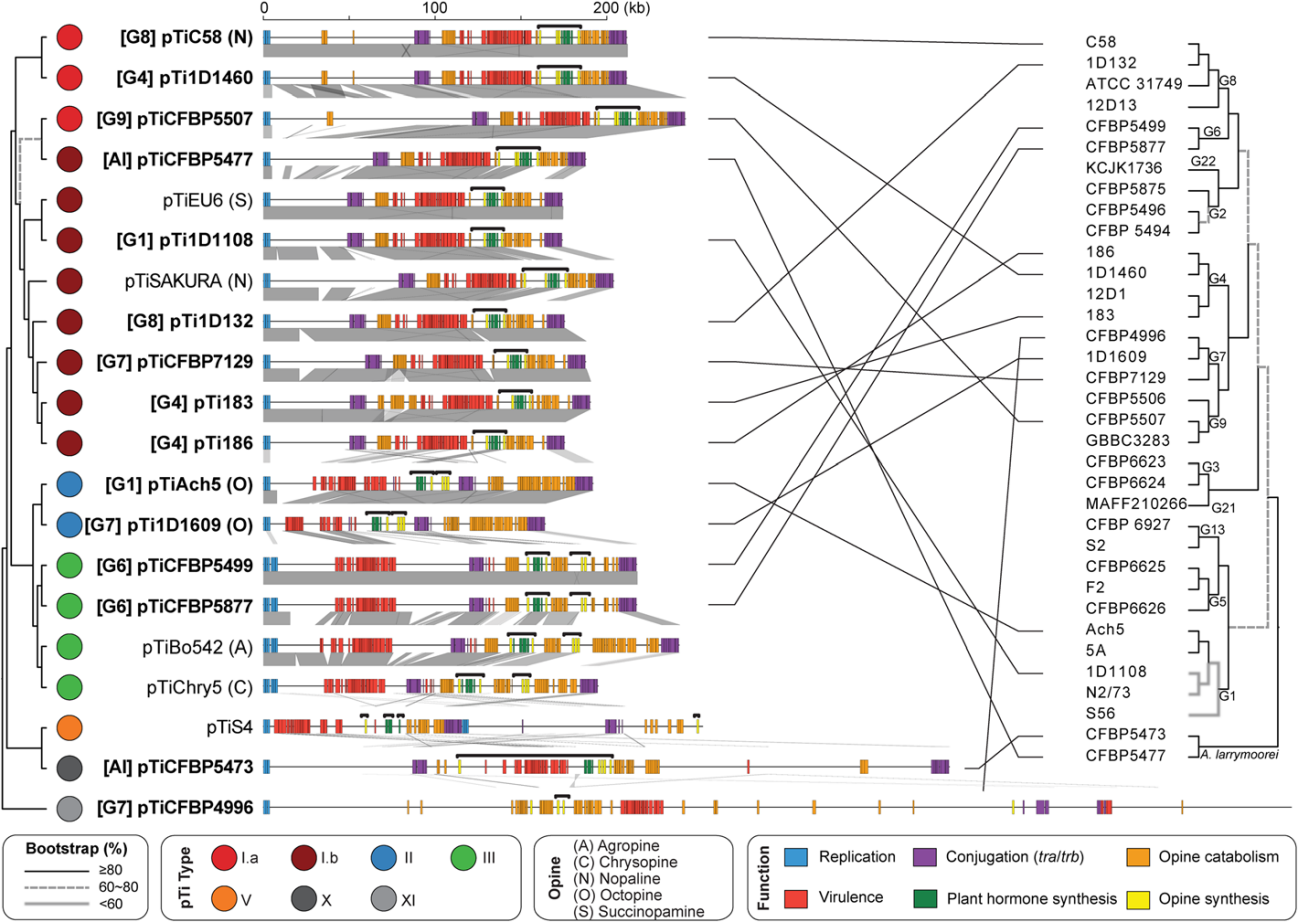
Molecular phylogeny and global alignment of pTi. The maximum likelihood phylogeny was inferred based on the concatenated alignment of 21 core genes and 8534 aligned amino acid sites (Supplementary Figure S6A). The species phylogeny on the right is based on Fig. 1. The pTi sequences derived from this study are highlighted in bold. For those with relevant information available, the genomospecies assignments are indicated in square brackets and the opine types are indicated in parentheses. For the alignment, all plasmids are visualized in linear form starting from the replication genes. The key gene clusters are color-coded according to functions, and the predicted T-DNA regions are indicated by the black horizontal bracket above each plasmid. Syntenic regions are indicated by gray blocks

The tumor-inducing plasmids (pTi) are an important component of *A. tumefaciens* genomes. These large accessory replicons harbor the virulence (*vir*) regulon genes that encode the Vir proteins and type IV secretion system (T4SS) for processing and delivering a transfer DNA (T-DNA) into plant cells, and are essential for agrobacterial phytopathogenicity [13–15]. Among the 35 strains examined, we identified 15 pTi sequences (Table 2). Two novel putative pTi (i.e., pTiCFBP4996 and pTiCFBP5473) were found in the 14 newly sequenced strains. This efficiency of discovering novel pTi types is surprising, given our previous study that defined pTi types I–VI was based on extensive sampling of diverse historical collections containing 162 *Agrobacterium* strains [9]. This finding demonstrates the importance and usefulness of a phylogeny-guided approach for investigating genetic diversity. Our recent examination of > 4000 Rhizobiaceae plasmids sampled from 1251 strains representing 222 species-level taxa assigned these two novel putative pTi to types X and XI [43]. Both types are rare; type X is found in CFBP5473 and only one other *A. larrymoorei* strain (AF3.44), CFBP4996 is the only strain that harbors a type XI plasmid. These two novel pTi are distinctive in their large sizes (Table 2), gene organization (Fig. 6), gene content (Additional file 1: Figure S6), and sequence divergence of core genes (Additional file 1: Figure S7). Moreover, their T-DNA regions also differ from the typical sizes of ~ 18–26 kb observed in types I–III pTi (Fig. 6). For the tumorigenic strain CFBP5473 (Additional file 1: Figure S8), the predicted T-DNA border sequences flank an exceptionally large (~ 93 kb) region that contains all of the *vir* regulon genes in addition to the typical T-DNA-associated genes (e.g., synthesis of opine and plant hormone) (Fig. 6). This reflects either a translocation of a T-DNA border sequence or reliance on a non-canonical T-DNA border sequence that we could not identify. For pTiCFBP4996, its 7-kb T-DNA is predicted to contain only four genes (i.e., two correspond to opine synthesis and two encode hypothetical proteins). Plant hormone synthesis genes, which are necessary to cause visible disease symptoms, were not identified in this predicted T-DNA region or elsewhere on this plasmid. Consistent with predictions, strain CFBP4996 did not induce tumor formation when inoculated onto stems of tomato plants (Additional file 1: Figure S8). Considering that pTiCFBP4996 encodes all essential *vir* genes required for T-DNA processing and transfer, CFBP4996 may serve as a naturally disarmed strain capable of T-DNA transfer without causing diseases.

**Table 2:** List of the pTi sequences analyzed. These include 15 from the genome data set listed in Table 1 and five additional representatives downloaded from GenBank. The pTi type assignments are based on k-mer profiles. A type V pTi from *Agrobacterium vitis* is included as the outgroup. Species name abbreviations: *At*, *Agrobacterium tumefaciens*; *Al*, *Agrobacterium larrymoorei*; *Av*, *Agrobacterium vitis*

Species	Strain	pTi	pTi type	Opine type	Accession	Size (bp)	Coding sequences	Geographic origin	Isolation source
*At* G1	1D1108	pTi1D1108	I.b	?	NZ_CP032925	176,213	159	MD, USA	*Euonymus* sp.
*At* G1	Ach5	pTiAch5	II	Octopine	NZ_CP011249	194,264	153	CA, USA	*Achillea ptarmica*
*At* G1	183	pTi183	I.b	?	NZ_CP029048	192,674	173	Tunisia	*Prunus dulcis*
*At* G4	186	pTi186	I.b	?	NZ_CP042277	177,577	159	CA, USA	*Juglans regia*
*At* G4	1D1460	pTi1D1460	I.a	?	NZ_CP032929	214,233	198	CA, USA	*Rubus* sp.
*At* G4	MAFF301001	pTiSAKURA	I.b	Nopaline	NC_002147	206,479	195	Japan	*Cerasus pseudocerasus*
*At* G6	CFBP5499	pTiCFBP5499	III	?	NZ_CP039893	220,025	178	South Africa	*Dahlia* sp.
*At* G6	CFBP5877	pTiCFBP5877	III	?	NZ_CP039902	220,025	181	Israel	*Dahlia* sp.
*At* G7	1D1609	pTi1D1609	II	Octopine	NZ_CP026926	166,117	138	CA, USA	*Medicago sativa*
*At* G7	CFBP4996	pTiCFBP4996	XI	?	NZ_CM016551	605,495	521	UK	*Flacourtia ramontchi*
*At* G7	CFBP7129	pTiCFBP7129	I.b	?	NZ_CP039927	189,955	176	Tunisia	*Pyrus communis*
*At* G8	1D132	pTi1D132	I.b	?	NZ_CP033026	177,577	160	CA, USA	*Cerasus pseudocerasus*
*At* G8	C58	pTiC58	I.a	Nopaline	NC_003065	214,233	197	NY, USA	*Cerasus pseudocerasus*
*At* G9	CFBP5507	pTiCFBP5507	I.a	?	NZ_CM016546	248,634	225	Australia	Soil
?	Bo542	pTiBo542	III	Agropine	NC_010929	244,978	223	Germany	*Dahlia* sp.
?	Chry5	pTiChry5	III	Chrysopine	KX388536	197,268	210	FL, USA	*Chrysanthemum* sp.
?	EU6	pTiEU6	I.b	Succinamopine	KX388535	176,375	194	CT, USA	*Euonymus* sp.
*Al*	CFBP5473	pTiCFBP5473	X	?	NZ_CP039694	404,101	360	FL, USA	*Ficus benjamina*
*Al*	CFBP5477	pTiCFBP5477	I.b	?	NZ_CM016547	190,036	169	Italy	?
*Av*	S4	pTiS4	V	Vitopine	NC_011982	258,824	193	Hungary	*Vitis* sp.

**Fig. 7:**
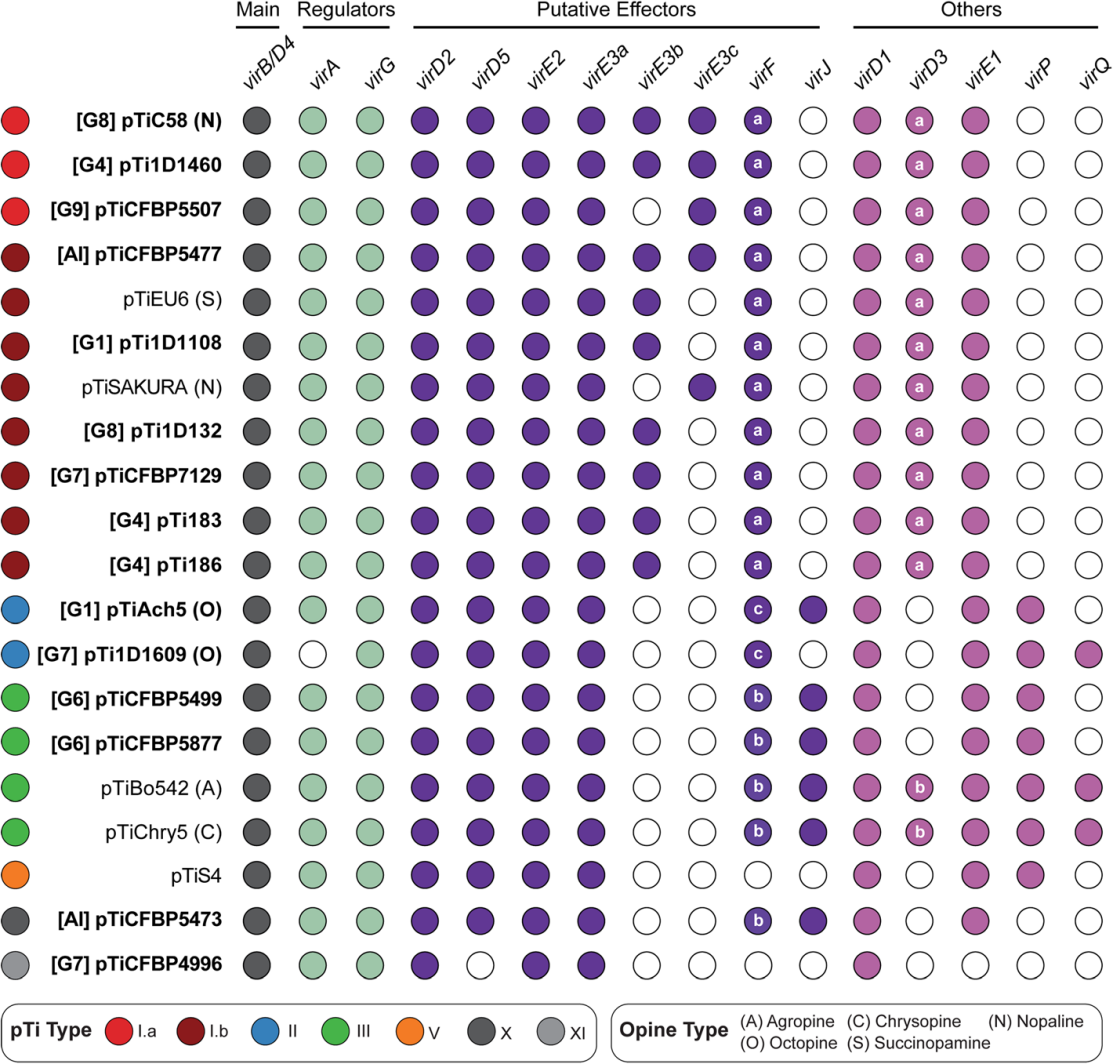
Distribution of key *vir* genes among the putative pTi. The pTi sequences derived from this study are highlighted in bold. For those with relevant information available, the genomospecies assignments are indicated in square brackets and the opine types are indicated in parentheses. Gene presence and absence are indicated by filled and empty circles, respectively. The main *vir* genes include the components of the type IV secretion system (T4SS; *virB1*-*B11* and *virD4*). For *virE3*, the three sequence types are listed separately. For *virF* and *virD3*, the sequence types are labeled inside the circles. For 1D1609, the genes *virA* and *virJ* are located on another plasmid and plotted as absent in this figure. The locus tags are provided in Supplementary Dataset S1B

**Fig. 8:**
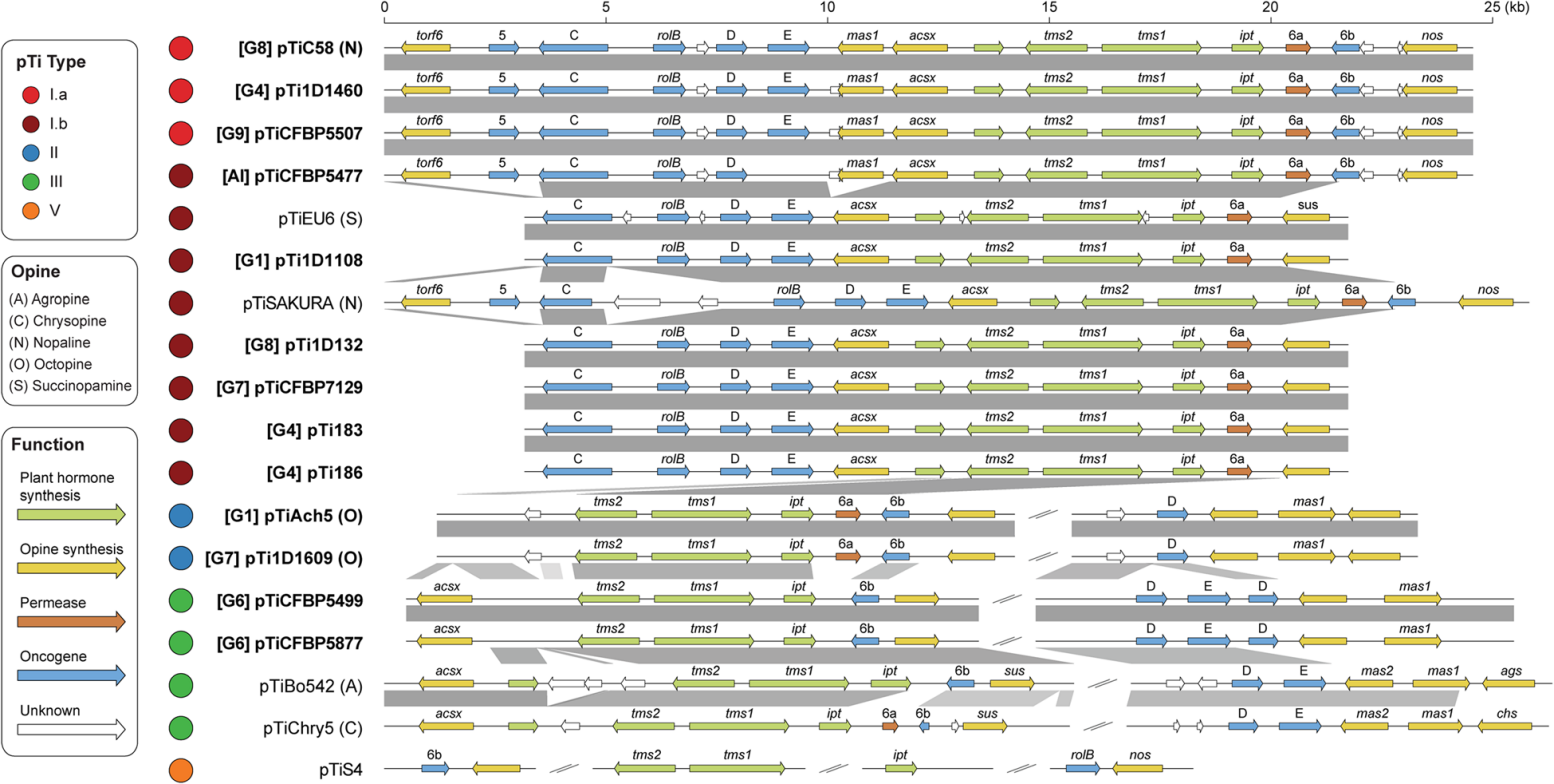
Organization of the transfer DNA (T-DNA) on tumor-inducing plasmids (pTi). Two unusual putative pTi sequences (i.e., pTiCFBP4996 and pTiCFBP5473) are excluded, and other pTi sequences derived from this study are highlighted in bold. For those with relevant information available, the genomospecies assignments are indicated in square brackets and the opine types are indicated in parentheses. Genes are color-coded according to annotation, syntenic regions are indicated by gray blocks

For replicon-level comparisons, types II and III pTi are more similar to each other than to type I pTi based on gene content (Additional file 1: Figure S6) and core gene phylogeny (Additional file 1: Figure S7). Within type I, the two subtypes (i.e., I.a and I.b) are distinguishable by gene content (Additional file 1: Figure S6) but do not form mutually exclusive clades in the core-gene phylogeny (Additional file 1: Figure S7). All putative pTi, including the two novel types and pTiS4 (i.e., type V from distantly-related *Agrobacterium vitis*), contain genes for the T4SS that mediates T-DNA transfer into plant cells (i.e., *virB1*-*B11* and *virD4*) and the corresponding two-component regulatory system (i.e., *virA* and *virG*) (Fig. 7). Strain 1D1609 is a notable case because its *virA* and *virJ* are located on another plasmid, rather than the pTi [26]. For the other *vir* regulon genes, several differences among pTi types were observed (Fig. 7). For example, while all pTi harbor a conserved *virE3a* that facilitates T-DNA protection and entry into host [44], type I pTi harbor one or two additional copies of *virE3* that belong to different sequence types (i.e., sharing the same annotation but classified as distinct homologs due to sequence divergence). Similarly, *virF*, which encodes an F-box protein that is a putative host-range determinant [45], can be classified into three sequence types with distinct distributions. Those less well-characterized *vir* genes, such as *virD3* [46, 47], *virJ* [48], and *virP*, are also distributed differently among pTi types. Finally, in addition to the presence/absence of individual genes, the overall organization of *vir* regulons also differ among these pTi (Additional file 1: Figure S9). All type I pTi are conserved in sharing a ~ 40-kb region that contains all *vir* genes. In comparison, locations of *vir* genes are more variable among type II/III pTi; *virF/P* (and *virQ/H* if present) are located ~ 5–50 kb away from the main *vir* gene cluster. Other than *vir* genes, the gene content and organization of T-DNA are also different (Fig. 8). All type I pTi have one single T-DNA region, while types II and III have two and type V have four, respectively [9] (Fig. 6). Within the T-DNA regions, the plant hormone synthesis genes (i.e., *tms1*/*iaaM*, *tms2/iaaH*, and *ipt*) are the most conserved ones, while others are more variable (Fig. 8). Taken together, this genetic variation may contribute to the host range differences observed among strains harboring different types of pTi. For example, based on a comparison among > 100 strains, there are strong associations between type I and III pTi with woody and herbaceous plants, respectively [9]. Moreover, previous tumorigenesis assays demonstrated that strains harboring types I and II pTi tend to have higher virulence against Brassicaceae and Asteraceae hosts, respectively [49].

## Discussion

### Biological entities at the species level and above

Based on the divergence of core gene sequences (Fig. 1A) and gene content (Additional file 1: Figure S1), 95% ANI is a reliable approach for defining species within the *A. tumefaciens* complex, as is the case for most other bacteria [10]. The discrete multimodal distribution of genome similarities (Fig. 1B) suggested that there are genetic barriers between different genomospecies, which may be explained by neutral processes and/or selection [8, 10]. Regardless of the exact mechanisms, these patterns supported application of the biological species concept, which is based on genetic barriers, to these *A. tumefaciens* genomospecies and other bacteria [3]. With the continuing drop in sequencing cost, ANI analysis can serve as a standard approach for accurate classification of additional strains [50], which in turn could facilitate research and communication, and ideally leads to improvements in bacterial taxonomy for basic works and applications.

It is worth noting that despite our effort, the 33 strains included in this study do not fully capture the diversity of the *A. tumefaciens* species complex. For example, the species *Agrobacterium arsenijevicii* [51], *Agrobacterium nepotum* (G14) [52], *Agrobacterium viscosum* (G15) [53], and two unnamed genomospecies G19/G20 [53] are also members of this species complex. As more strains are characterized in the future, it is likely that higher levels of diversity within this group will continue to be discovered. Furthermore, several potentially confusing issues regarding *Agrobacterium* taxonomy remain to be resolved. For example, strain MAFF210266 that we referred to as a representative of G21 shares 98% ANI with an important strain K599 (=NCPPB2659) [54]. However, K599 harbors a root-inducing plasmid (pRi) associated with hairy root disease and was named as *Agrobacterium rhizogenes* (a.k.a. *Agrobacterium* biovar 2 [12] or G10 [16], all deprecated synonyms for *Rhizobium rhizogenes*), which is phylogenetically divergent from the *A. tumefaciens* species complex. Recently, during the revision of this work, the name *Agrobacterium* G21 was independently proposed and strain K599 was reclassified to G21 [55].This example highlights the dynamic nature of pTi/pRi transmission within the agrobacteria-rhizobia complex [9] and advocates a classification scheme based on genome-wide ANI, rather than plasmid content or phenotype.

At the above-species level, *A. tumefaciens* genomospecies exhibited some intriguing patterns of genome divergence. In a previous study that compared ~ 90,000 prokaryotic genomes, it was extremely rare to find ANI values in the range of 82–96% [10]. In other words, strains either belong to the same natural biological entity at the species level and have > 95% ANI, or belong to different species and have < 82% ANI. This observation could be due to biases in the sampling of available genomes, or the all-against-all pairwise comparisons included mostly distantly-related species [11]. In our study that provided a detailed examination of closely related species, the ~ 85–93% ANI among *A. tumefaciens* genomospecies (Fig. 1B) indicated that the *A. tumefaciens* complex is indeed a coherent entity with high divergence from its closest sister lineage within the same genus (i.e., *A. larrymoorei*). The driving forces for maintaining species complexes and the prevalence of such above-species level entities are interesting questions that require further investigations. For the *A. tumefaciens* complex, although the nomenclature originated from its phytopathogenicity, it is well-established that this group contains both pathogenic and non-pathogenic strains that differ in the possession of an oncogenic plasmid (i.e., pTi) or not [12]. The promiscuous nature of their pTi [9, 56–58] (Fig. 6) suggested that lineages within this complex may experience frequent transitions between pathogenic and non-pathogenic lifestyles, and such shared ecological niches may be the force that maintains the coherence of this species complex. Compared to sister lineages (e.g., *A. larrymoorei* and *A. rubi*), the more diverse host range of *A. tumefaciens* [12, 13] may be linked to the higher diversity of pTi types [9], which may have facilitated the divergence of this complex into multiple genomospecies. To test this hypothesis, better sampling of these sister lineages is required.

At the genus level and above, genome-based classification is more challenging. The ANI approach is expected to have limited resolution when nucleotide sequence identities are below ~ 80% [10]. Moreover, the fractions of genome sequences alignable for ANI value calculation are highly variable for genus-level comparisons [59], which raises concerns on the robustness of applying the ANI method to higher taxonomic ranks. To resolve this challenge, analysis of protein sequence divergence among core genes was proposed as a suitable approach [60]. However, while it may be desirable to establish a standardized taxonomy with a defined range of genomic divergence for each taxonomic rank, large variations in the divergence values at a given rank were observed among different taxonomic groups in previous attempts [59–61]. These variations created situations where some families contain higher divergence levels than some orders or lower divergence levels than some genera, even after normalization and a full revision of the current taxonomy [60]. Such situations demonstrated the challenges of establishing a new standardized taxonomy even when the practical issues of transitioning from the current taxonomy are not considered, and perhaps is to be expected given the highly variable evolutionary rates across different lineages [62]. Given these considerations, other aspects of biology (e.g., physiology, ecology) may play more important roles in defining those higher taxonomic ranks.

### Units and modularity of molecular evolution

For evolutionary studies, the levels at which selection and other processes operate on have been a topic that received much attention [63]. For prokaryotes, levels from the entire genome to individual functional domains within genes are of particular interest. Based on our results, all of these levels must be considered to comprehend the complex patterns.

At the whole-genome level, the clear species boundaries based on overall similarity (Fig. 1 and Additional file 1: Figure S1) suggested that the entire genome largely evolves as a single coherent unit. This result is consistent with previous findings that at the global level HGT has very little impact on the reconstruction of organismal phylogeny [64, 65], despite the extensive HGT inferred in bacterial evolution [65–68] and the importance of HGT in adaptation [69–71]. A possible explanation for these seemingly conflicting observations is that most of the acquired genes are lost quickly [70], presumably due to the strong mutational bias towards deletions observed in bacteria [72, 73]. Additionally, acquired genes are subjected to the selection that drives species diversification [8], which is expected to act on all genes in a genome together.

At the level of individual replicons, chromosomes and plasmids certainly have distinct evolutionary histories (Fig. 6). Because novel chromosome/plasmid combinations may lead to speciation [57], and the spread of plasmids has important implications on the evolution of virulence [9, 74] and antimicrobial resistance [75], further investigations on the evolution of plasmids and their compatibilities with chromosomes are important [9, 76, 77]. Additionally, for bacteria with multiple chromosomes, examining the evolution of individual chromosomes may provide novel insights. In the case of *A. tumefaciens*, the multipartite genome was hypothesized to originate from intragenomic gene transfer from the ancestral circular chromosome to a plasmid, followed by linearization of this plasmid to form the secondary chromosome [23, 78]. The secondary chromosome is known to exhibit higher levels of divergence in overall organization, gene content, and sequences [23, 26]. In this regard, it is curious to note that the apparently rapid-evolving T6SS genes are all located on the secondary chromosome, rather than the more conserved primary chromosome. For future studies, it may be interesting to compare the molecular evolution of T6SS and other genes between species with mono- and multi-partite genomes.

At the levels of gene clusters and below, several interesting observations were made based on the loci of the two secretion systems investigated in this work. First, although these two systems may provide some fitness advantages (e.g., T6SS for interbacterial competitions and T4SS for host exploitation), complex patterns of gains and losses were observed (Figs. 2 and 6). These patterns suggest that there is not a strong selective pressure to maintain these genes and non-adaptive stochastic processes are important. Alternatively, these genes may be subjected to heterogeneous selective pressures. Regardless, there is a certain degree of modularity regarding their evolution, such that the presence patterns are all-or-none and no partial cluster was found for the chromosomal T6SS genes or the plasmid-encoded T4SS genes. This is particularly evident for the T6SS genes, as when the main cluster was lost (e.g., G2 and G6), no accessory *vgrG* loci located elsewhere was found (Additional file 1: Figure S3). Second, for both systems, genes for the structural components are conserved, while those for the effectors and others are not (Figs. 2, 5, and 7). This is consistent with the expectation that opposite selective forces may act on these two categories of genes, with purifying selection against changes to preserve the apparatus of a functional secretion system and positive selection for more diverse effectors and other accessory components. Third, modularity that may reflect functional constraints were observed at finer scales. For example, each *vgrG* is linked to its cognate effector/chaperone genes, and each effector is linked to its cognate immunity gene. Such modularity is expected to be maintained by selection, similar to the observations regarding co-transfers of genes involved in associated biochemical pathways [8]. However, the linkage could be broken down by recombination at within- or between-species levels, as evident in the diversity of *vgrG* gene neighborhoods, even for those homologs belonging to the same type (Fig. 4). Fourth, at the level of individual genes, the within-genome diversity of *vgrG* homologs (Fig. 5) provides further support to the hypothesis that HGT is more important than duplications in driving gene family expansions in bacteria [79]. Finally, at the intra-gene level, within- or between-species recombination may be important in generating novel combinations of domains, thus promoting the diversification of homologs (Fig. 3 and Additional file 1: Figure S6).

Taken together, these observations illustrated the complexity of biological systems. While it is difficult to draw up generalized rules or to estimate the relative importance of each evolutionary process at different levels, it is important to consider and examine these complexities to better understand organisms of interest.

## Conclusions

In summary, by using a group of important bacteria as the study system, this work utilized a strategy of phylogeny-conscious genome sampling for systematic investigations of a species complex. This approach requires prior knowledge regarding the extant phylogenetic diversity of the study system, which is important in the planning stage of large-scale genome-sequencing projects. In addition to improving the cost-effectiveness, this strategy is also critical in obtaining an unbiased picture that does not over-emphasize certain subgroups. Furthermore, the emphasis on generating and utilizing high-quality assemblies improves the confidence in gene content analysis. With the continuing advancements in sequencing technologies and bioinformatic tools, this emphasis becomes increasingly accessible. For this study system, our examination of biological boundaries at the species level and above improves the understanding of how natural biodiversity is organized. The targeted analysis of those secretion system genes and oncogenic plasmids provides novel insights regarding the key genetic variations involved in the fitness and ecology of these soil-borne phytopathogens that need to compete in complex microbiota and invade plant hosts. Moreover, the multi-level analysis of their genetic diversity from whole-genome to intra-genic domains highlights the complexity of these biological systems. The strategy and findings of this work provide useful guides for future studies of other bacteria.

## Methods

### Genome sequencing

A total of 14 strains were acquired from the French International Center for Microbial Resources (CIRM) Collection for Plant-associated Bacteria (CFBP) (Table 1). These include 12 strains that belong to the *A. tumefaciens* species complex and two *A. larrymoorei* strains as the outgroup.

The procedure for whole-genome shotgun sequencing was based on that described in our previous studies [7, 80, 81]. All bioinformatics tools were used with the default settings unless stated otherwise. Briefly, total genomic DNA was prepared using the Wizard Genomic DNA purification kit (Promega, USA). The Illumina paired-end sequencing libraries were prepared using KAPA LTP Library Preparation Kits (Roche Sequencing, USA) with a targeted insert size of ~ 550 bp. The Illumina MiSeq platform was used to generate 300 × 2 reads with an average coverage of 306-fold per strain (range 141- to 443-fold). The raw reads were quality trimmed using a Q20 cutoff and used for de novo assembly based on Velvet v1.2.10 [82] with the settings “-exp_cov auto -min_contig_lgth 2000 -scaffolding no.” The contigs were oriented by mapping to those complete genome assemblies available (Table 1) using MAUVE v2015-02-13 [83]. Due to the difficulties of identifying appropriate reference genomes for several evolutionary branches, four strains (i.e., CFBP5473, CFBP5875, CFBP5877, and CFBP6623) were selected for PacBio long-read sequencing and PacBio HGAP v3 assembly. These PacBio-based assemblies were used as a guide for scaffolding, rather than the finalized results.

To improve the Illumina-based draft assemblies, an iterative process was used to examine the raw reads mapping results and to incorporate gap-filling results based on PCR and Sanger sequencing. This process was repeated until the complete assembly was obtained or the draft assembly could not be improved further. The finalized assemblies were submitted to the National Center for Biotechnology Information (NCBI) and annotated using the Prokaryotic Genome Annotation Pipeline (PGAP) [84].

### Comparative and evolutionary analysis

The genomes analyzed are listed in Table 1. The procedures for genome comparisons were based on those described in our previous studies [7, 85–87]. Briefly, pairwise genome similarities were calculated using FastANI v1.1 [10]. For comparisons of plasmids, FastANI was executed with the custom settings that reduced fragment length to 1000 bp and minimum matched fragments to 25. For global alignments of chromosomes and plasmids, the syntenic regions were identified by BLASTN v2.6.0 [34] and visualized using genoPlotR v0.8.9 [88]. For gene content comparison, BLASTP v2.6.0 [34] with e-value cutoff set to 1e^−15^ and OrthoMCL v1.3 [89] were used to infer the homologous gene clusters. The result was converted into a matrix of 35 genomes by 17,058 clusters, with the value in each cell corresponding to the copy number. This matrix was further converted into a Jaccard distance matrix among genomes using the VEGAN package v2.5-6 in R, then processed using the principal coordinates analysis function in the APE package [90] and visualized using ggplot2 v3.3.2 [91]. The hierarchical clustering analysis was performed using PVCLUST v3.4.4 [92].

For phylogenetic analysis, homologous sequences were aligned using MUSCLE v3.8.31 [93] for maximum likelihood inference by PhyML v.3.3.20180214 [94]. The proportion of invariable sites and the gamma distribution parameter were estimated from the data set, and the number of substitute rate categories was set to four. The bootstrap supports were estimated based on 1000 replicates.

### Analysis of the type VI secretion system genes

To identify the T6SS-associated genes, C58 [27, 36, 37] and other strains [7, 33] that have been characterized experimentally were used as the references. Based on the known T6SS genes in these genomes, homologous genes in other genomes were identified based on the OrthoMCL result. To screen for novel T6SS effector, chaperone, and immunity genes, genes that are located near *vgrG* (i.e., three upstream and ten downstream) were examined manually by using the NCBI conserved domain database (CDD) [95] and the Phyre2 protein fold recognition server [96]; the e-value cutoff was set to 0.01. A few genes with a hit to the DUF4123 domain (i.e., a known T6SS chaperone) but have an e-value above the cutoff were manually added back to the list of T6SS-associated genes (e.g., CFBP5477_RS20350). To confirm the absence of specific T6SS genes, the genome sequences were used as the subjects and the protein sequences of known genes were used as the queries to run TBLASTN searches.

For classification of the *vgrG* homologs, we developed a domain-based scheme. The conserved N-terminal TIGR03361 domain was first identified by the NCBI CDD searches. A global alignment of all homologs was used to determine the exact boundaries of this domain. After this TIGR03361 domain was removed, the remaining C-terminal sequences were processed using MEME v5.1.1 [97] to identify conserved domains that meet these criteria: (1) present in at least two sequences, (2) with zero or one occurrence per sequence, (3) with a size between 30 and 300 a.a., and (4) with an e-value lower than 0.05. The results were manually curated to break down large domains that are composed of smaller domains. Pairwise BLASTP searches were conducted to verify that each domain is unique and no two domains have a BLASTP e-value of lower than 1e−05. For each domain, the consensus sequence was generated using Jalview v2.10.5 [98] and sequence conservation was visualized using WebLogo server v3 [99]. For functional prediction, the consensus sequence of each domain was used to query against NCBI CDD and Phyre2. Additionally, one representative from each subtype of *vgrG* homologs was used for structure modeling using Phyre2 with the “normal” mode. The chain D of PA0091 VgrG1 (PDB identifier: 4MTK) was selected as the template. The predicted structures were visualized using PyMOL v1.2r3pre (Schrödinger, USA).

For the EI gene pairs identified, EI1 through EI11 were named based on the nomenclature proposed previously [7], and novel pairs were named starting from EI12. When only the putative effector (E) or the putative immunity (I) genes were found, those genes were classified in the format of “E??” or “I??”, respectively. For some of the EI pairs that were identified previously based on adjacency to T6SS genes but lacked high-confidence annotation (i.e., EI2, EI3, EI5, and EI8), we chose a more conservative approach and annotated those genes as hypothetical proteins.

### Analysis of the tumor-inducing plasmids and type IV secretion system genes

The list of 20 putative pTi sequences analyzed is provided in Table 2. These included all of the 15 complete sequences determined in this study and five representatives from GenBank that are important in *Agrobacterium* research [15]. Our definition of putative pTi was based on the presence of the main T4SS genes (*virB1*-*B11* and *virD4*) and at least one predicted T-DNA region. The pTi typing was performed based on k-mer profile clustering with a reference set of 143 oncogenic plasmids in Rhizobiaceae [9] and a second set that contains > 4000 Rhizobiaceae plasmids [43]. For T-DNA identification, putative T-DNA borders were identified based on the motif YGRCAGGATATATNNNNNKGTMAWN [100]. Genes involved in opine metabolism [101] and T4SS [102] were identified based on the annotation and homologous gene clustering results produced by OrthoMCL. Additionally, putative T4SS effectors were identified using the T4SEpre tool [103] in EffectiveDB [104] with the minimal score set to 0.8. All protein sequences of pTi-encoded genes were used as the queries.

### Tumorigenesis assay

Tomato tumorigenesis assays [105] were performed to evaluate the virulence of selected strains. The plants (cultivar Known-You 301) were maintained in growth chambers with a 16-/8-h light/dark regime and a constant temperature of 22 °C. Inoculation was performed on 3-week-old seedlings. Bacterial strains were transferred from stock to 5 mL 523 broth and cultured overnight at 28 °C in a shaker incubator (250 rpm), then sub-cultured for 4 h prior to inoculation. Bacterial cells were washed and resuspended in 0.9% NaCl solution with a concentration of OD_600_ 0.2. The stem was punctured with a sterilized sewing needle, and 5 μL of bacterial suspension was added to the wounding site. The plants were collected 3 weeks after inoculation and 1-cm stem segments centered at the wounding site were cut for weighing.

## Supplementary Information

**Additional file 1: Figure S1.** Gene content dissimilarity among the *Agrobacterium* genomes. (A) and (B): principal coordinate analysis with and without the outgroup *A. larrymoorei*, respectively. The % variance explained by each axis is provided in parentheses. (C) and (D): hierarchical clustering with and without the outgroup *A. larrymoorei*, respectively. **Figure S2.** Global alignment of the linear chromosomes. Locations of T6SS-*hcp* operons and *vgrG* homologs are labeled. **Figure S3.** Logo plots of the putative protein domains identified among *vgrG* homologs. For each domain, the length and the number of homologs with the domain is labeled. Domain 1 is the only domain with a corresponding database entry (TIGR03361). **Figure S4.** Predicted structures of VgrG homologs. Regions are colored according to the scheme used in the domain analysis (Fig. 3). The chain D of PA0091 VgrG1 (PDB identifier: 4MTK) from *Pseudomonas aeruginosa* was selected as the template. The C-terminal parts that could not be confidently inferred are omitted. In all cases, the coverage (i.e., percentage of the sequence included in the structure prediction) are at least 75%, the sequence identity to the template is at least 30% and the confidence score is 100%. **Figure S5.** Maximum likelihood phylogenies of *vgrG*-associated domains. (A) Domain 1 (TIGR03361; VI_Rhs_Vgr super family), (B) Domain 2 (unknown function), and (C) Domain 3 (unknown function). **Figure S6.** Principal coordinate analysis of gene content among the putative pTi analyzed. **Figure S7.** Maximum likelihood phylogeny of pTi based on the concatenated alignment of shared single-copy genes. (A) All of the 20 pTi sequences analyzed; 21 core genes and 8534 aligned amino acid sites. (B) Excluding the two novel pTi; 40 core genes and 15,473 aligned amino acid sites, all branches received > 80% bootstrap support. **Figure S8.** Tomato tumor assay of strains 12D1, CFBP4996, and CFBP5473. Mock was inoculated with sterilized water as a negative control and strain C58 was included as a positive control. Strain 12D1 harbors a plasmid with opine transporter and catabolism genes but lacks *vir* regulon genes and identifiable T-DNA. CFBP4996 and CFBP5473 harbor novel types of putative tumor-inducing plasmids (pTi). (A) Tomato stems at three weeks after inoculation. Scale bar: 0.25 cm. (B) Weight distribution of five biological replicates (1-cm segments of the stem centered at the inoculation site). The letters indicate ANOVA results. **Figure S9.** Gene organization of the *vir* regulons on pTi. Syntenic regions are indicated by grey blocks. The virulence (*vir*) genes are highlighted in red, the conjugation (*tra*) genes are highlighted in yellow, and other genes are plotted in white.

**Additional file 2: Dataset S1.** List of *vgrG*-associated genes. Information including genomic location, RefSeq annotation, and domain prediction are included. **Dataset S2.** Locus tags of the *vir* regulon genes on pTi. The *virA*/*J* of 1D1609 are located on another plasmid and are highlighted by “*”. The *virB7* of pTiChry5 and pTiEU6 are unannotated in the GenBank RefSeq records so no locus tag is available but the gene presence was confirmed by BLASTN searches.

## Data Availability

The 14 new genome sequences are available in NCBI under BioProject accessions PRJNA534385-PRJNA534397 and PRJNA534399.

## References

[1] Rosselló-Mora R, Amann R (2001). The species concept for prokaryotes. FEMS Microbiol Rev..

[2] Fraser C, Alm EJ, Polz MF, Spratt BG, Hanage WP (2009). The bacterial species challenge: making sense of genetic and ecological diversity. Science..

[3] Bobay L-M, Ochman H (2017). Biological species are universal across life’s domains. Genome Biol Evol..

[4] Konstantinidis K, Ramette A, Tiedje JM (2006). The bacterial species definition in the genomic era. Philos Trans R Soc B Biol Sci..

[5] Popoff MY, Kersters K, Kiredjian M, Miras I, Coynault C (1984). Position taxonomique de souches de *Agrobacterium* d’origine hospitalière. Ann Inst Pasteur Microbiol..

[6] Costechareyre D, Bertolla F, Nesme X (2009). Homologous recombination in *Agrobacterium*: potential implications for the genomic species concept in bacteria. Mol Biol Evol..

[7] Wu C-F, Santos MNM, Cho S-T, Chang H-H, Tsai Y-M, Smith DA (2019). Plant-pathogenic *Agrobacterium tumefaciens* strains have diverse type VI effector-immunity pairs and vary in in-planta competitiveness. Mol Plant Microbe Interact..

[8] Lassalle F, Planel R, Penel S, Chapulliot D, Barbe V, Dubost A (2017). Ancestral genome estimation reveals the history of ecological diversification in *Agrobacterium*. Genome Biol Evol..

[9] Weisberg AJ, Davis EW, Tabima J, Belcher MS, Miller M, Kuo C-H (2020). Unexpected conservation and global transmission of agrobacterial virulence plasmids. Science..

[10] Jain C, Rodriguez-R LM, Phillippy AM, Konstantinidis KT, Aluru S (2018). High throughput ANI analysis of 90 K prokaryotic genomes reveals clear species boundaries. Nat Commun..

[11] Murray CS, Gao Y, Wu M (2021). Re-evaluating the evidence for a universal genetic boundary among microbial species. Nat Commun..

[12] Young JM, Tzfira T, Citovsky V (2008). Agrobacterium—taxonomy of plant-pathogenic *Rhizobium* species. *Agrobacterium* Biol Biotechnol.

[13] Kado CI (2014). Historical account on gaining insights on the mechanism of crown gall tumorigenesis induced by *Agrobacterium tumefaciens*. Front Microbiol..

[14] Nester EW (2015). *Agrobacterium*: nature’s genetic engineer. Front Plant Sci..

[15] Hwang H-H, Yu M, Lai E-M (2017). *Agrobacterium*-mediated plant transformation: biology and applications. Arab Book..

[16] Mougel C, Thioulouse J, Perrière G, Nesme X (2002). A mathematical method for determining genome divergence and species delineation using AFLP. Int J Syst Evol Microbiol..

[17] Portier P, Saux MF-L, Mougel C, Lerondelle C, Chapulliot D, Thioulouse J (2006). Identification of genomic species in *Agrobacterium* biovar 1 by AFLP genomic markers. Appl Environ Microbiol..

[18] Costechareyre D, Rhouma A, Lavire C, Portier P, Chapulliot D, Bertolla F (2010). Rapid and efficient identification of *Agrobacterium* species by *recA* allele analysis: *Agrobacterium recA* diversity. Microb Ecol..

[19] Hellens R, Mullineaux P, Klee H (2000). A guide to *Agrobacterium* binary Ti vectors. Trends Plant Sci..

[20] Lee L-Y, Gelvin SB (2008). T-DNA binary vectors and systems. Plant Physiol..

[21] Lassalle F, Campillo T, Vial L, Baude J, Costechareyre D, Chapulliot D (2011). Genomic species are ecological species as revealed by comparative genomics in *Agrobacterium tumefaciens*. Genome Biol Evol..

[22] Young JM, Pennycook SR, Watson DRW (2006). Proposal that *Agrobacterium radiobacter* has priority over *Agrobacterium tumefaciens*. Request for an Opinion. Int J Syst Evol Microbiol..

[23] Slater SC, Goldman BS, Goodner B, Setubal JC, Farrand SK, Nester EW (2009). Genome sequences of three *Agrobacterium* biovars help elucidate the evolution of multichromosome genomes in bacteria. J Bacteriol..

[24] Goodner B, Hinkle G, Gattung S, Miller N, Blanchard M, Qurollo B (2001). Genome sequence of the plant pathogen and biotechnology agent *Agrobacterium tumefaciens* C58. Science..

[25] Wood DW, Setubal JC, Kaul R, Monks DE, Kitajima JP, Okura VK (2001). The genome of the natural genetic engineer *Agrobacterium tumefaciens* C58. Science..

[26] Haryono M, Cho S-T, Fang M-J, Chen A-P, Chou S-J, Lai E-M (2019). Differentiations in gene content and expression response to virulence induction between two *Agrobacterium* strains. Front Microbiol..

[27] Ma L-S, Hachani A, Lin J-S, Filloux A, Lai E-M (2014). Agrobacterium tumefaciens deploys a superfamily of type VI secretion DNase effectors as weapons for interbacterial competition *in planta*. Cell Host Microbe..

[28] Benson DA, Cavanaugh M, Clark K, Karsch-Mizrachi I, Ostell J, Pruitt KD (2018). GenBank. Nucleic Acids Res..

[29] Ormeño-Orrillo E, Servín-Garcidueñas LE, Rogel MA, González V, Peralta H, Mora J (2015). Taxonomy of rhizobia and agrobacteria from the Rhizobiaceae family in light of genomics. Syst Appl Microbiol..

[30] Hernandez RE, Gallegos-Monterrosa R, Coulthurst SJ (2020). Type VI secretion system effector proteins: effective weapons for bacterial competitiveness. Cell Microbiol..

[31] Jurėnas D, Journet L (2021). Activity, delivery, and diversity of type VI secretion effectors. Mol Microbiol..

[32] Smith WPJ, Vettiger A, Winter J, Ryser T, Comstock LE, Basler M (2020). The evolution of the type VI secretion system as a disintegration weapon. PLoS Biol..

[33] Santos MNM, Cho S-T, Wu C-F, Chang C-J, Kuo C-H, Lai E-M (2020). Redundancy and specificity of type VI secretion *vgrG* loci in antibacterial activity of *Agrobacterium tumefaciens* 1D1609 strain. Front Microbiol..

[34] Camacho C, Coulouris G, Avagyan V, Ma N, Papadopoulos J, Bealer K (2009). BLAST+: architecture and applications. BMC Bioinformatics..

[35] Wu H-Y, Chung P-C, Shih H-W, Wen S-R, Lai E-M (2008). Secretome analysis uncovers an Hcp-family protein secreted via a type VI secretion system in *Agrobacterium tumefaciens*. J Bacteriol..

[36] Bondage DD, Lin J-S, Ma L-S, Kuo C-H, Lai E-M (2016). VgrG C terminus confers the type VI effector transport specificity and is required for binding with PAAR and adaptor–effector complex. Proc Natl Acad Sci..

[37] Lin J-S, Ma L-S, Lai E-M (2013). Systematic dissection of the *Agrobacterium* type VI secretion system reveals machinery and secreted components for subcomplex formation. PLoS One..

[38] Pukatzki S, Ma AT, Revel AT, Sturtevant D, Mekalanos JJ (2007). Type VI secretion system translocates a phage tail spike-like protein into target cells where it cross-links actin. Proc Natl Acad Sci..

[39] Leiman PG, Basler M, Ramagopal UA, Bonanno JB, Sauder JM, Pukatzki S (2009). Type VI secretion apparatus and phage tail-associated protein complexes share a common evolutionary origin. Proc Natl Acad Sci..

[40] Wu C-F, Weisberg AJ, Davis EW, Chou L, Khan S, Lai E-M (2021). Diversification of the type VI secretion system in agrobacteria. mBio..

[41] Liang X, Moore R, Wilton M, Wong MJQ, Lam L, Dong TG (2015). Identification of divergent type VI secretion effectors using a conserved chaperone domain. Proc Natl Acad Sci..

[42] Unterweger D, Kostiuk B, Ötjengerdes R, Wilton A, Diaz-Satizabal L, Pukatzki S (2015). Chimeric adaptor proteins translocate diverse type VI secretion system effectors in *Vibrio cholerae*. EMBO J..

[43] Weisberg AJ, Miller M, Ream W, Grünwald NJ, Chang JH (2022). Diversification of plasmids in a genus of pathogenic and nitrogen-fixing bacteria. Philos Trans R Soc B Biol Sci.

[44] Li X, Tu H, Pan SQ (2018). *Agrobacterium* delivers anchorage protein VirE3 for companion VirE2 to aggregate at host entry sites for T-DNA protection. Cell Rep..

[45] Jarchow E, Grimsley NH, Hohn B (1991). virF, the host-range-determining virulence gene of *Agrobacterium tumefaciens*, affects T-DNA transfer to *Zea mays*. Proc Natl Acad Sci..

[46] Vogel AM, Das A (1992). The *Agrobacterium tumefaciens virD3* gene is not essential for tumorigenicity on plants. J Bacteriol..

[47] Lin T-S, Kado CI (1993). The *virD4* gene is required for virulence while *virD3* and *orf5* are not required for virulence of *Agrobacterium tumefaciens*. Mol Microbiol..

[48] Pan SQ, Jin S, Boulton MI, Hawes M, Gordon MP, Nester EW (1995). An *Agrobacterium* virulence factor encoded by a Ti plasmid gene or a chromosomal gene is required for T-DNA transfer into plants. Mol Microbiol..

[49] Hwang H-H, Wu ET, Liu S-Y, Chang S-C, Tzeng K-C, Kado CI (2013). Characterization and host range of five tumorigenic *Agrobacterium tumefaciens* strains and possible application in plant transient transformation assays. Plant Pathol..

[50] de Lajudie PM, Andrews M, Ardley J, Eardly B, Jumas-Bilak E, Kuzmanović N (2019). Minimal standards for the description of new genera and species of rhizobia and agrobacteria. Int J Syst Evol Microbiol..

[51] Kuzmanović N, Puławska J, Prokić A, Ivanović M, Zlatković N, Jones JB (2015). *Agrobacterium arsenijevicii* sp. nov., isolated from crown gall tumors on raspberry and cherry plum. Syst Appl Microbiol..

[52] Mousavi SA, Willems A, Nesme X, de Lajudie P, Lindström K (2015). Revised phylogeny of Rhizobiaceae: proposal of the delineation of *Pararhizobium* gen. nov., and 13 new species combinations. Syst Appl Microbiol..

[53] Mafakheri H, Taghavi SM, Puławska J, de Lajudie P, Lassalle F, Osdaghi E (2019). Two novel genomospecies in the *Agrobacterium tumefaciens* species complex associated with rose crown gall. Phytopathology..

[54] Valdes Franco JA, Collier R, Wang Y, Huo N, Gu Y, Thilmony R (2016). Draft genome sequence of *Agrobacterium rhizogenes* strain NCPPB2659. Genome Announc..

[55] Singh NK, Lavire C, Nesme J, Vial L, Nesme X, Mason CE, et al. Comparative genomics of novel *Agrobacterium* G3 strains isolated from the International Space Station and description of *Agrobacterium tomkonis* sp. nov. Front Microbiol. 2021;12:3369. 10.3389/fmicb.2021.765943 PMC8685578 34938279

[56] Hooykaas PJJ, Klapwijk PM, Nuti MP, Schilperoort RA, Rörsch A (1977). Transfer of the *Agrobacterium tumefaciens* Ti plasmid to avirulent agrobacteria and to Rhizobium ex planta. J Gen Microbiol..

[57] Haryono M, Tsai Y-M, Lin C-T, Huang F-C, Ye Y-C, Deng W-L (2018). Presence of an *Agrobacterium*-type tumor-inducing plasmid in Neorhizobium sp. NCHU2750 and the link to phytopathogenicity. Genome Biol Evol..

[58] Rathore DS, Mullins E, Roberts JA (2018). Alternative non-*Agrobacterium* based methods for plant transformation. Annu Plant Rev Online.

[59] Barco RA, Garrity GM, Scott JJ, Amend JP, Nealson KH, Emerson D (2020). A genus definition for Bacteria and Archaea based on a standard genome relatedness index. mBio..

[60] Parks DH, Chuvochina M, Waite DW, Rinke C, Skarshewski A, Chaumeil P-A (2018). A standardized bacterial taxonomy based on genome phylogeny substantially revises the tree of life. Nat Biotechnol..

[61] Parks DH, Chuvochina M, Chaumeil P-A, Rinke C, Mussig AJ, Hugenholtz P (2020). A complete domain-to-species taxonomy for Bacteria and Archaea. Nat Biotechnol..

[62] Kuo C-H, Ochman H (2009). Inferring clocks when lacking rocks: the variable rates of molecular evolution in bacteria. Biol Direct..

[63] Okasha S. Evolution and the Levels of Selection. Oxford: Oxford University Press; 2006. Available from: https://oxford.universitypressscholarship.com/view/10.1093/acprof:oso/9780199267972.001.0001/acprof-9780199267972

[64] Daubin V, Moran NA, Ochman H (2003). Phylogenetics and the cohesion of bacterial genomes. Science..

[65] Choi I-G, Kim S-H (2007). Global extent of horizontal gene transfer. Proc Natl Acad Sci..

[66] Ochman H, Lawrence JG, Groisman EA (2000). Lateral gene transfer and the nature of bacterial innovation. Nature..

[67] Dagan T, Artzy-Randrup Y, Martin W (2008). Modular networks and cumulative impact of lateral transfer in prokaryote genome evolution. Proc Natl Acad Sci..

[68] Chan CX, Beiko RG, Darling AE, Ragan MA (2009). Lateral transfer of genes and gene fragments in prokaryotes. Genome Biol Evol..

[69] Pál C, Papp B, Lercher MJ (2005). Adaptive evolution of bacterial metabolic networks by horizontal gene transfer. Nat Genet..

[70] Kuo C-H, Ochman H (2009). The fate of new bacterial genes. FEMS Microbiol Rev..

[71] Wiedenbeck J, Cohan FM (2011). Origins of bacterial diversity through horizontal genetic transfer and adaptation to new ecological niches. FEMS Microbiol Rev..

[72] Mira A, Ochman H, Moran NA (2001). Deletional bias and the evolution of bacterial genomes. Trends Genet..

[73] Kuo C-H, Ochman H (2009). Deletional bias across the three domains of life. Genome Biol Evol..

[74] Sundin GW (2007). Genomic insights into the contribution of phytopathogenic bacterial plasmids to the evolutionary history of their hosts. Annu Rev Phytopathol..

[75] Bennett PM (2009). Plasmid encoded antibiotic resistance: acquisition and transfer of antibiotic resistance genes in bacteria. Br J Pharmacol..

[76] Smillie C, Garcillán-Barcia MP, Francia MV, Rocha EPC, de la Cruz F (2010). Mobility of plasmids. Microbiol Mol Biol Rev..

[77] Redondo-Salvo S, Fernández-López R, Ruiz R, Vielva L, de Toro M, Rocha EPC (2020). Pathways for horizontal gene transfer in bacteria revealed by a global map of their plasmids. Nat Commun..

[78] Ramírez-Bahena MH, Vial L, Lassalle F, Diel B, Chapulliot D, Daubin V (2014). Single acquisition of protelomerase gave rise to speciation of a large and diverse clade within the *Agrobacterium*/*Rhizobium* supercluster characterized by the presence of a linear chromid. Mol Phylogenet Evol..

[79] Treangen TJ, Rocha EPC (2011). Horizontal transfer, not duplication, drives the expansion of protein families in prokaryotes. PLoS Genet..

[80] Huang Y-Y, Cho S-T, Lo W-S, Wang Y-C, Lai E-M, Kuo C-H (2015). Complete genome sequence of *Agrobacterium tumefaciens* Ach5. Genome Announc..

[81] Cho S-T, Haryono M, Chang H-H, Santos MNM, Lai E-M, Kuo C-H (2018). Complete genome sequence of *Agrobacterium tumefaciens* 1D1609. Genome Announc..

[82] Zerbino DR, Birney E (2008). Velvet: algorithms for *de novo* short read assembly using de Bruijn graphs. Genome Res..

[83] Darling ACE, Mau B, Blattner FR, Perna NT (2004). Mauve: multiple alignment of conserved genomic sequence with rearrangements. Genome Res..

[84] Tatusova T, DiCuccio M, Badretdin A, Chetvernin V, Nawrocki EP, Zaslavsky L (2016). NCBI prokaryotic genome annotation pipeline. Nucleic Acids Res..

[85] Lo W-S, Chen L-L, Chung W-C, Gasparich GE, Kuo C-H (2013). Comparative genome analysis of *Spiroplasma melliferum* IPMB4A, a honeybee-associated bacterium. BMC Genomics..

[86] Lo W-S, Gasparich GE, Kuo C-H (2018). Convergent evolution among ruminant-pathogenic *Mycoplasma* involved extensive gene content changes. Genome Biol Evol..

[87] Cho S-T, Kung H-J, Huang W, Hogenhout SA, Kuo C-H (2020). Species boundaries and molecular markers for the classification of 16SrI phytoplasmas inferred by genome analysis. Front Microbiol..

[88] Guy L, Roat Kultima J, Andersson SGE (2010). genoPlotR: comparative gene and genome visualization in R. Bioinformatics..

[89] Li L, Stoeckert CJ, Roos DS (2003). OrthoMCL: identification of ortholog groups for eukaryotic genomes. Genome Res..

[90] Popescu A-A, Huber KT, Paradis E (2012). ape 3.0: New tools for distance-based phylogenetics and evolutionary analysis in R. Bioinformatics..

[91] Wickham H (2016). ggplot2: Elegant Graphics for Data Analysis.

[92] Suzuki R, Shimodaira H (2006). Pvclust: an R package for assessing the uncertainty in hierarchical clustering. Bioinformatics..

[93] Edgar RC (2004). MUSCLE: multiple sequence alignment with high accuracy and high throughput. Nucleic Acids Res..

[94] Guindon S, Gascuel O (2003). A simple, fast, and accurate algorithm to estimate large phylogenies by maximum likelihood. Syst Biol..

[95] Marchler-Bauer A, Zheng C, Chitsaz F, Derbyshire MK, Geer LY, Geer RC (2013). CDD: conserved domains and protein three-dimensional structure. Nucleic Acids Res..

[96] Kelley LA, Mezulis S, Yates CM, Wass MN, Sternberg MJE (2015). The Phyre2 web portal for protein modeling, prediction and analysis. Nat Protoc..

[97] Bailey TL, Boden M, Buske FA, Frith M, Grant CE, Clementi L (2009). MEME Suite: tools for motif discovery and searching. Nucleic Acids Res..

[98] Waterhouse AM, Procter JB, Martin DMA, Clamp M, Barton GJ (2009). Jalview version 2—a multiple sequence alignment editor and analysis workbench. Bioinformatics..

[99] Crooks GE, Hon G, Chandonia J-M, Brenner SE (2004). WebLogo: a sequence logo generator. Genome Res..

[100] Conner AJ, Barrell PJ, Baldwin SJ, Lokerse AS, Cooper PA, Erasmuson AK (2007). Intragenic vectors for gene transfer without foreign DNA. Euphytica..

[101] Vladimirov IA, Matveeva TV, Lutova LA (2015). Opine biosynthesis and catabolism genes of *Agrobacterium tumefaciens* and *Agrobacterium rhizogenes*. Russ J Genet..

[102] Bhatty M, Laverde Gomez JA, Christie PJ (2013). The expanding bacterial type IV secretion lexicon. Res Microbiol..

[103] Wang Y, Wei X, Bao H, Liu S-L (2014). Prediction of bacterial type IV secreted effectors by C-terminal features. BMC Genomics..

[104] Eichinger V, Nussbaumer T, Platzer A, Jehl M-A, Arnold R, Rattei T (2016). EffectiveDB—updates and novel features for a better annotation of bacterial secreted proteins and Type III, IV, VI secretion systems. Nucleic Acids Res.

[105] Wu H-Y, Chen C-Y, Lai E-M (2014). Expression and functional characterization of the *Agrobacterium* VirB2 amino acid substitution variants in T-pilus biogenesis, virulence, and transient transformation efficiency. PLoS One..

