# Supplementary material for: Modular evolution of secretion systems and virulence plasmids in a bacterial species complex
Source: BMC Biol. 2022 Jan 13;20:16. doi: 10.1186/s12915-021-01221-y (PMC8756689; doi:10.1186/s12915-021-01221-y)
Supplement: Supplementary file 1 — Additional file 1: Figure S1. Gene content dissimilarity among the Agrobacterium genomes. (A) and (B): principal coordinate analysis with and without the outgroup A. larrymoorei, respectively. The % variance explained by each axis is provided in parentheses. (C) and (D): hierarchical clustering with and without the outgroup A. larrymoorei, respectively. Figure S2. Global alignment of the linear chromosomes. Locations of T6SS-hcp operons and vgrG homologs are labeled. Figure S3. Logo plots of the putative protein domains identified among vgrG homologs. For each domain, the length and the number of homologs with the domain is labeled. Domain 1 is the only domain with a corresponding database entry (TIGR03361). Figure S4. Predicted structures of VgrG homologs. Regions are colored according to the scheme used in the domain analysis (Fig. 3). The chain D of PA0091 VgrG1 (PDB identifier: 4MTK) from Pseudomonas aeruginosa was selected as the template. The C-terminal parts that could not be confidently inferred are omitted. In all cases, the coverage (i.e., percentage of the sequence included in the structure prediction) are at least 75%, the sequence identity to the template is at least 30% and the confidence score is 100%. Figure S5. Maximum likelihood phylogenies of vgrG-associated domains. (A) Domain 1 (TIGR03361; VI_Rhs_Vgr super family), (B) Domain 2 (unknown function), and (C) Domain 3 (unknown function). Figure S6. Principal coordinate analysis of gene content among the putative pTi analyzed. Figure S7. Maximum likelihood phylogeny of pTi based on the concatenated alignment of shared single-copy genes. (A) All of the 20 pTi sequences analyzed; 21 core genes and 8534 aligned amino acid sites. (B) Excluding the two novel pTi; 40 core genes and 15,473 aligned amino acid sites, all branches received > 80% bootstrap support. Figure S8. Tomato tumor assay of strains 12D1, CFBP4996, and CFBP5473. Mock was inoculated with sterilized water as a negative control and strain C5 [file 12915_2021_1221_MOESM1_ESM.pdf]

Supplementary Information

Additional File 1: Figures S1-S9

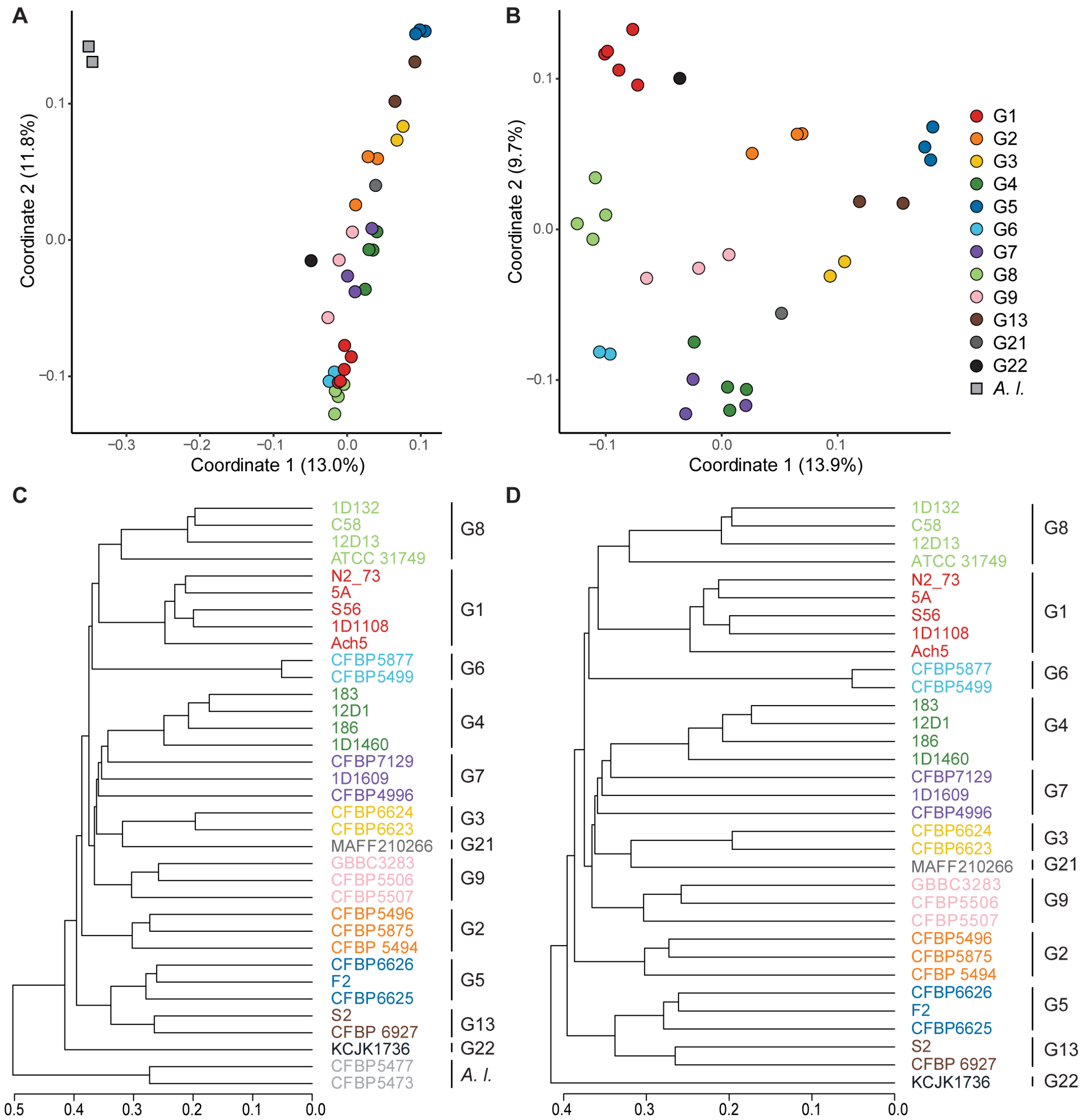

Figure S1. Gene content dissimilarity among the *Agrobacterium* genomes. (A) and (B): principal coordinate analysis with and without the outgroup *A. larrymoorei*, respectively. The % variance explained by each axis is provided in parentheses. (C) and (D): hierarchical clustering with and without the outgroup *A. larrymoorei*, respectively.

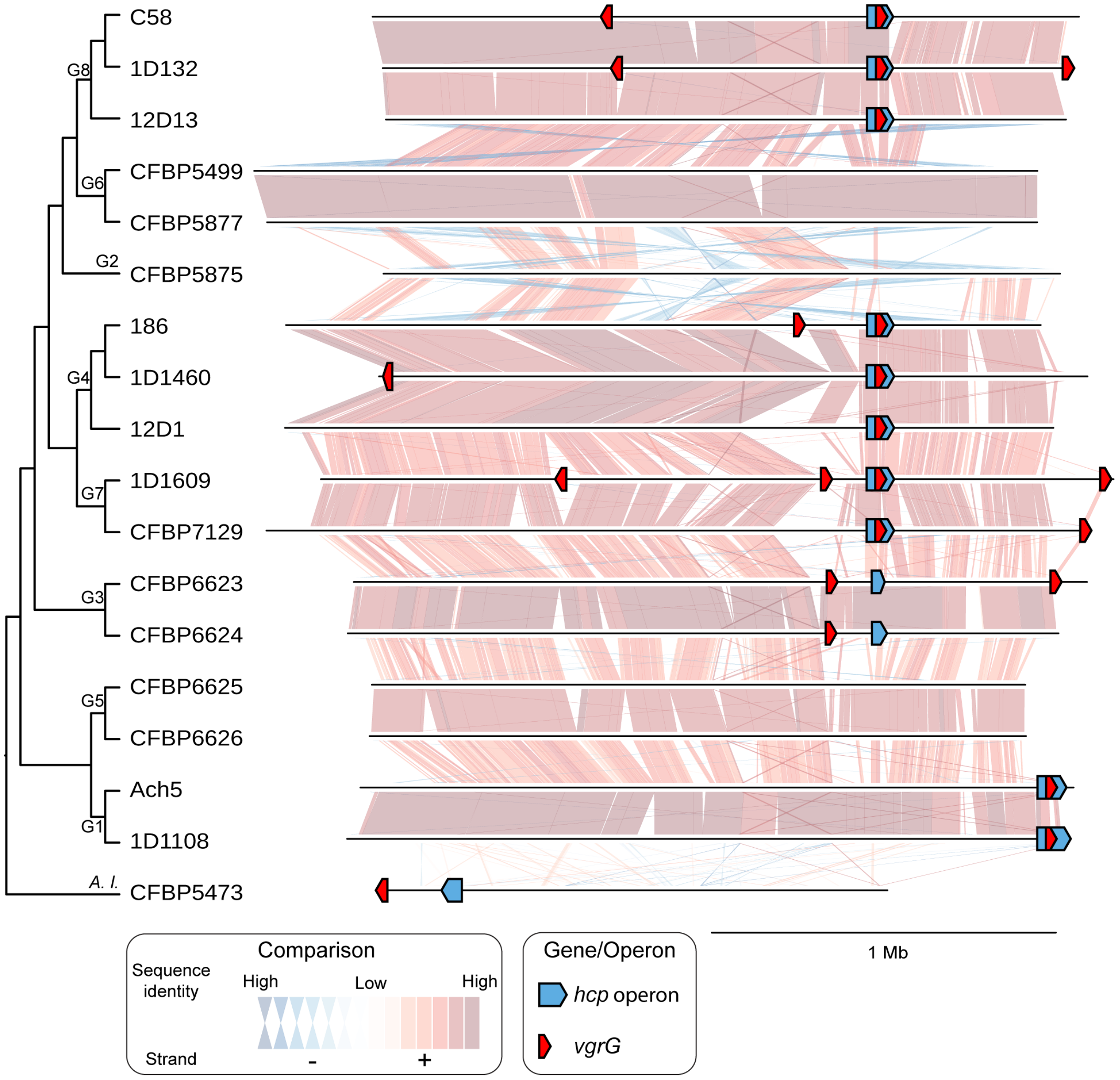

Figure S2. Global alignment of the linear chromosomes. Locations of T6SS-*hcp* operons and *vgrG* homologs are labeled.

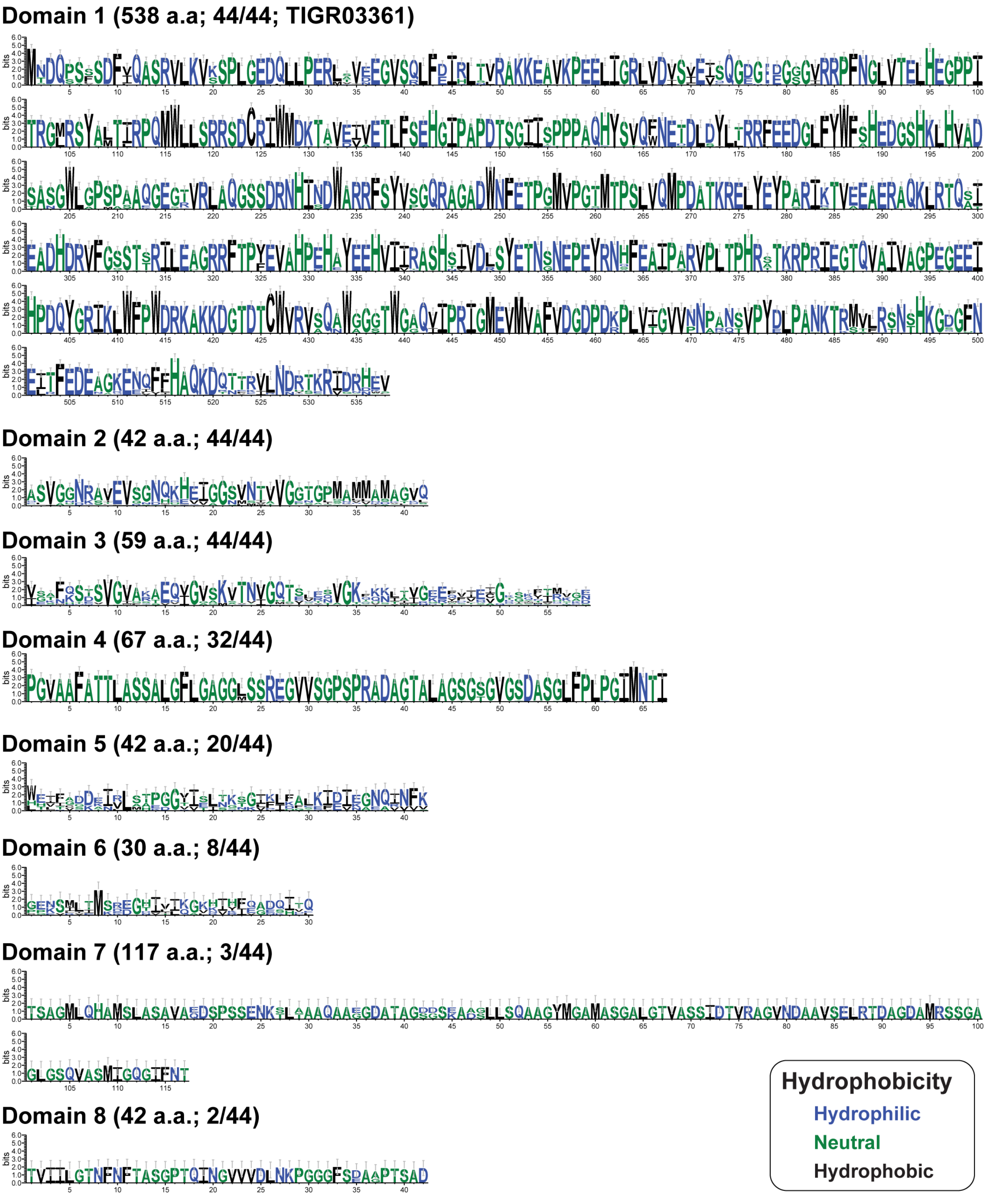

Figure S3. Logo plots of the putative protein domains identified among *vgrG* homologs. For each domain, the length and the number of homologs with the domain is labeled. Domain 1 is the only domain with a corresponding database entry (TIGR03361).

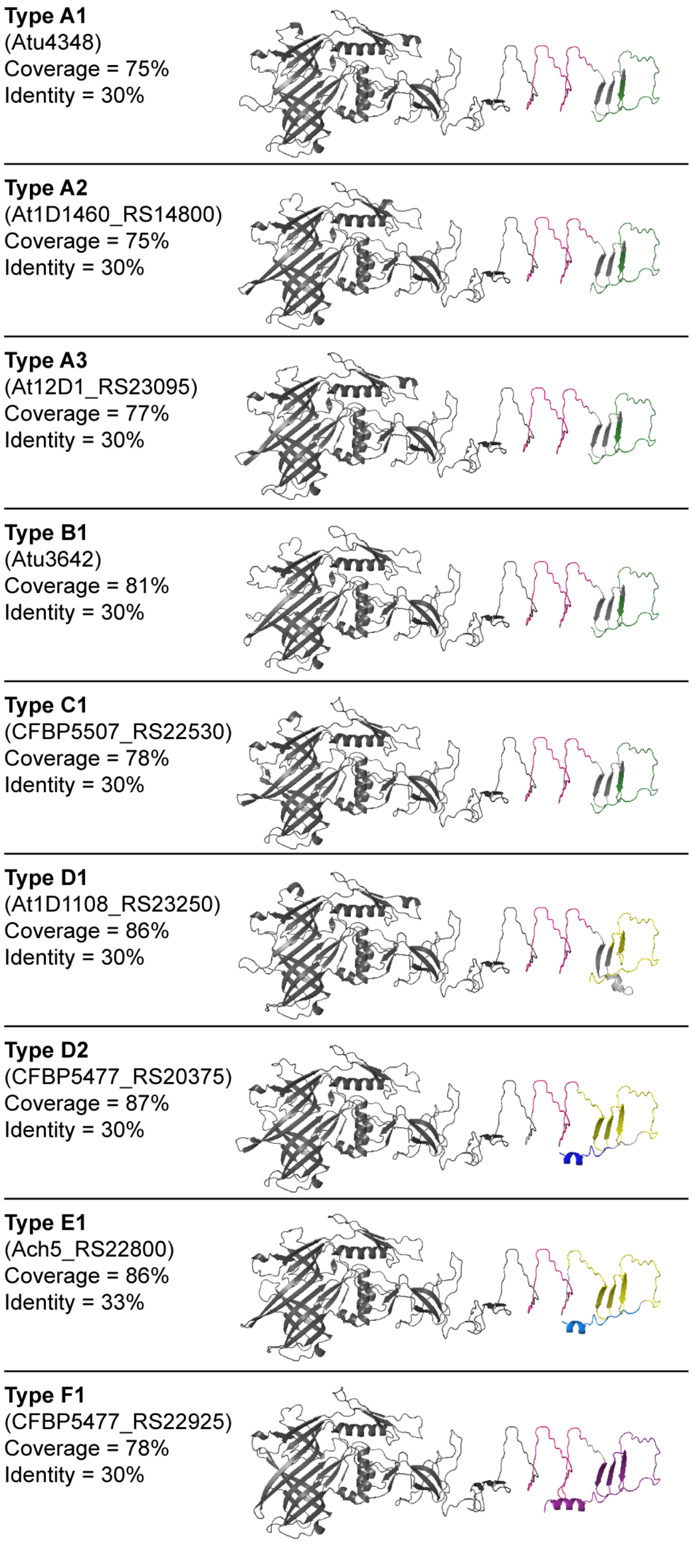

Figure S4. Predicted structures of VgrG homologs. Regions are colored according to the scheme used in the domain analysis (**Figure. 3**). The chain D of PA0091 VgrG1 (PDB identifier: 4MTK) from *Pseudomonas aeruginosa* was selected as the template. The C-terminal parts that could not be confidently inferred are omitted. In all cases, the coverage (i.e., percentage of the sequence included in the structure prediction) are at least 75%, the sequence identity to the template is at least 30% and the confidence score is 100%.

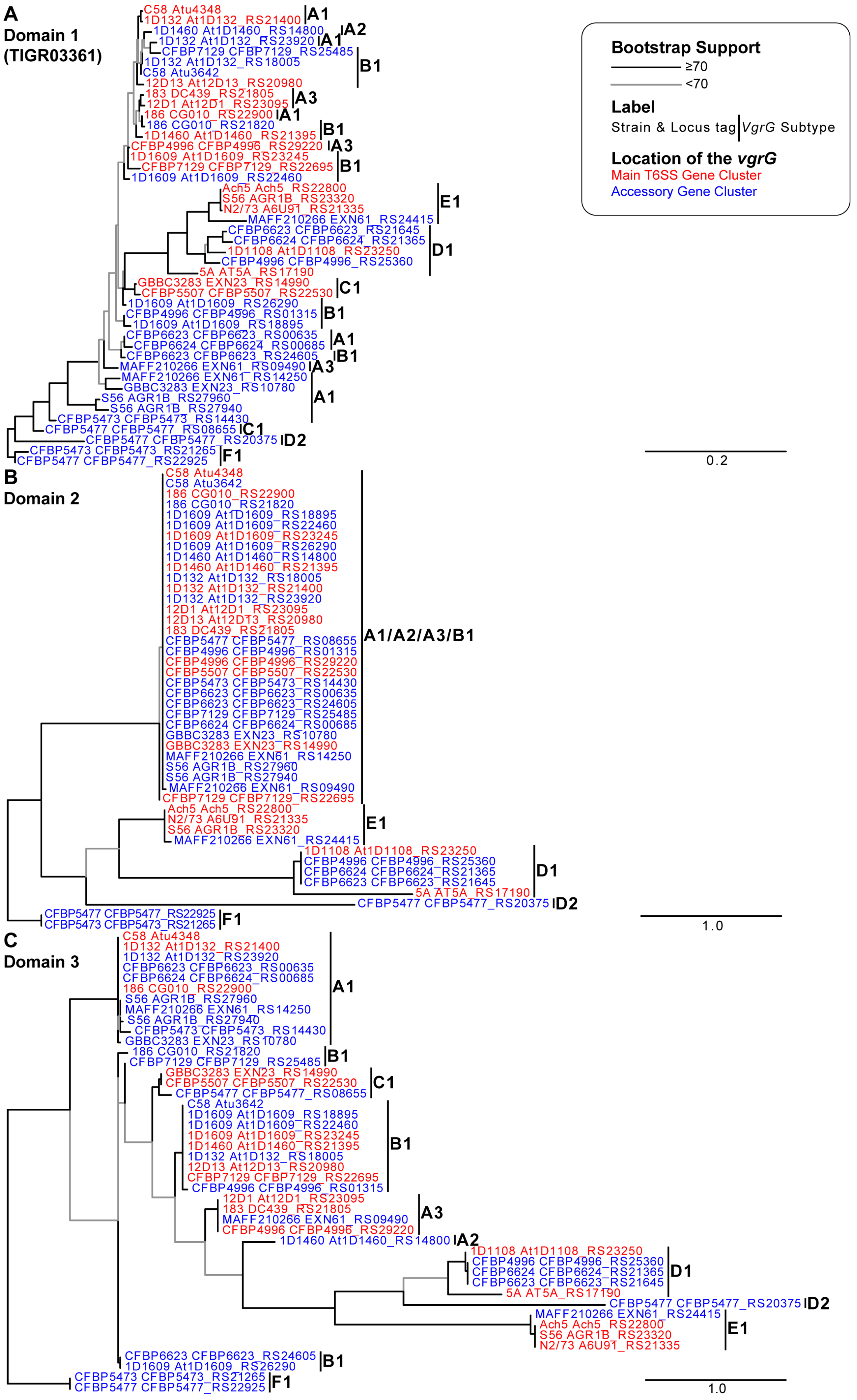

Figure S5. Maximum likelihood phylogenies of *vgrG*-associated domains. (A) Domain 1 (TIGR03361; VI_Rhs_Vgr super family), (B) Domain 2 (unknown function), and (C) Domain 3 (unknown function).

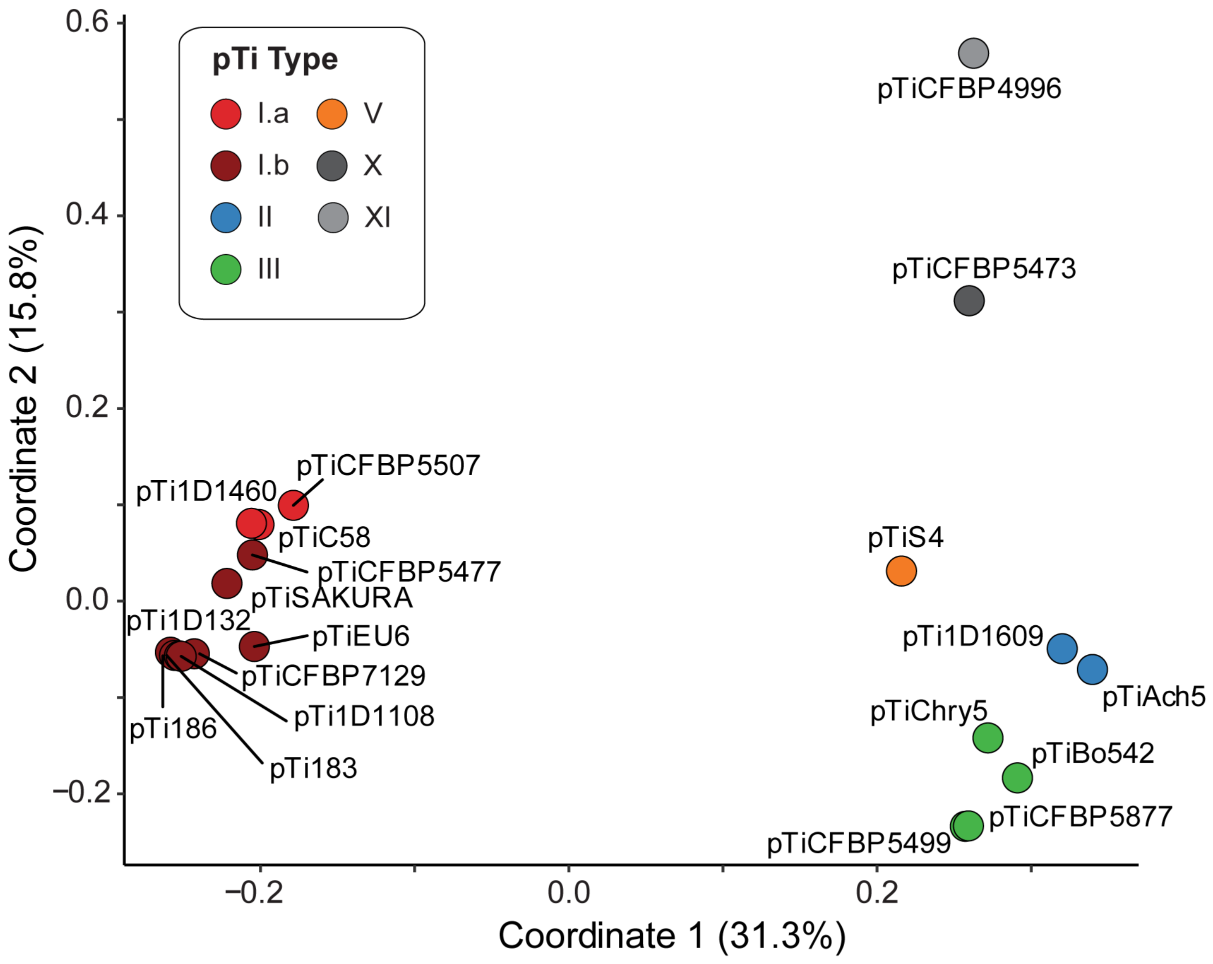

Figure S6. Principal coordinate analysis of gene content among the putative pTi analyzed.

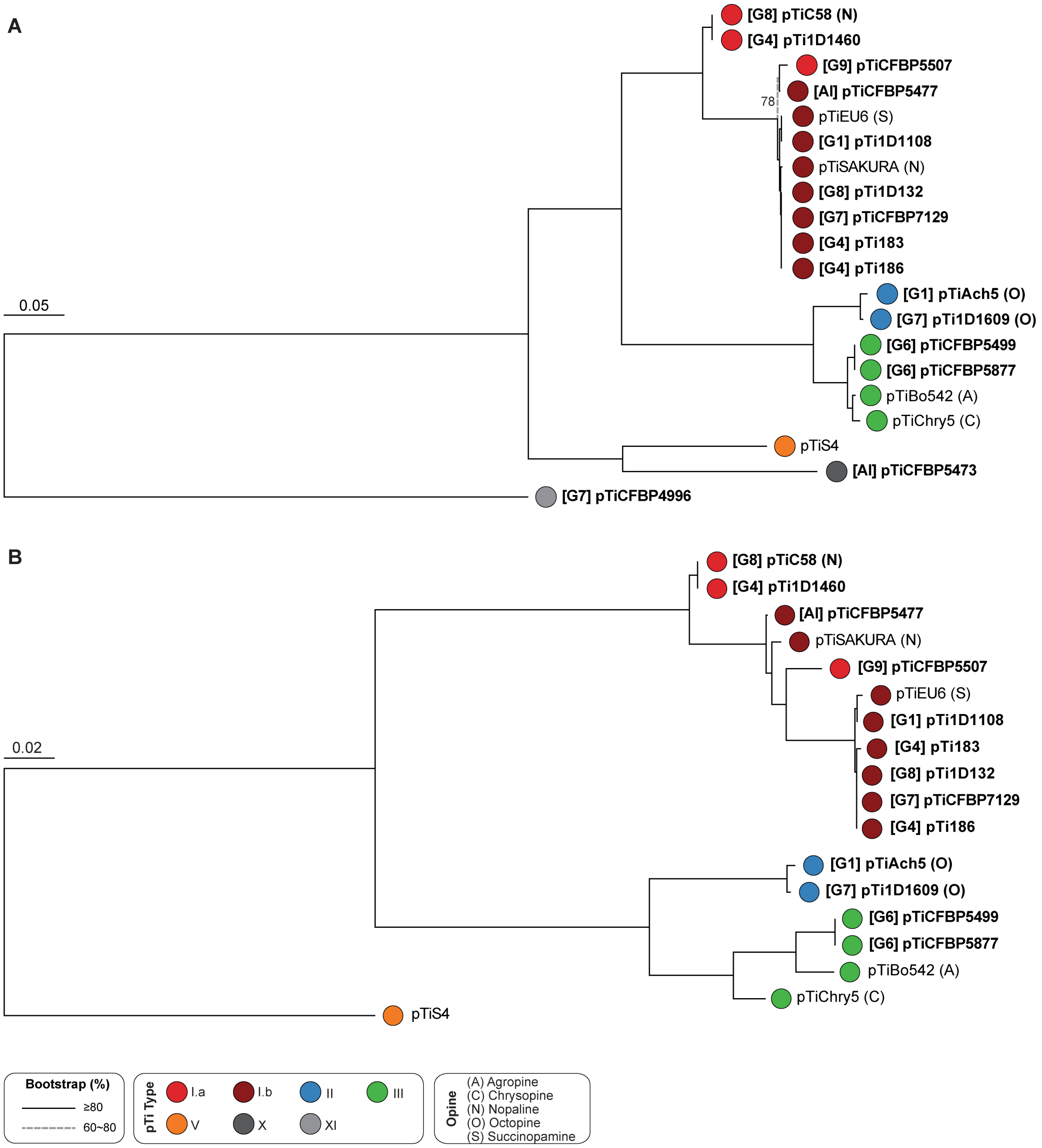

Figure S7. Maximum likelihood phylogeny of pTi based on the concatenated alignment of shared single-copy genes. (A) All of the 20 pTi sequences analyzed; 21 core genes and 8,534 aligned amino acid sites. (B) Excluding the two novel pTi; 40 core genes and 15,473 aligned amino acid sites, all branches received > 80% bootstrap support.

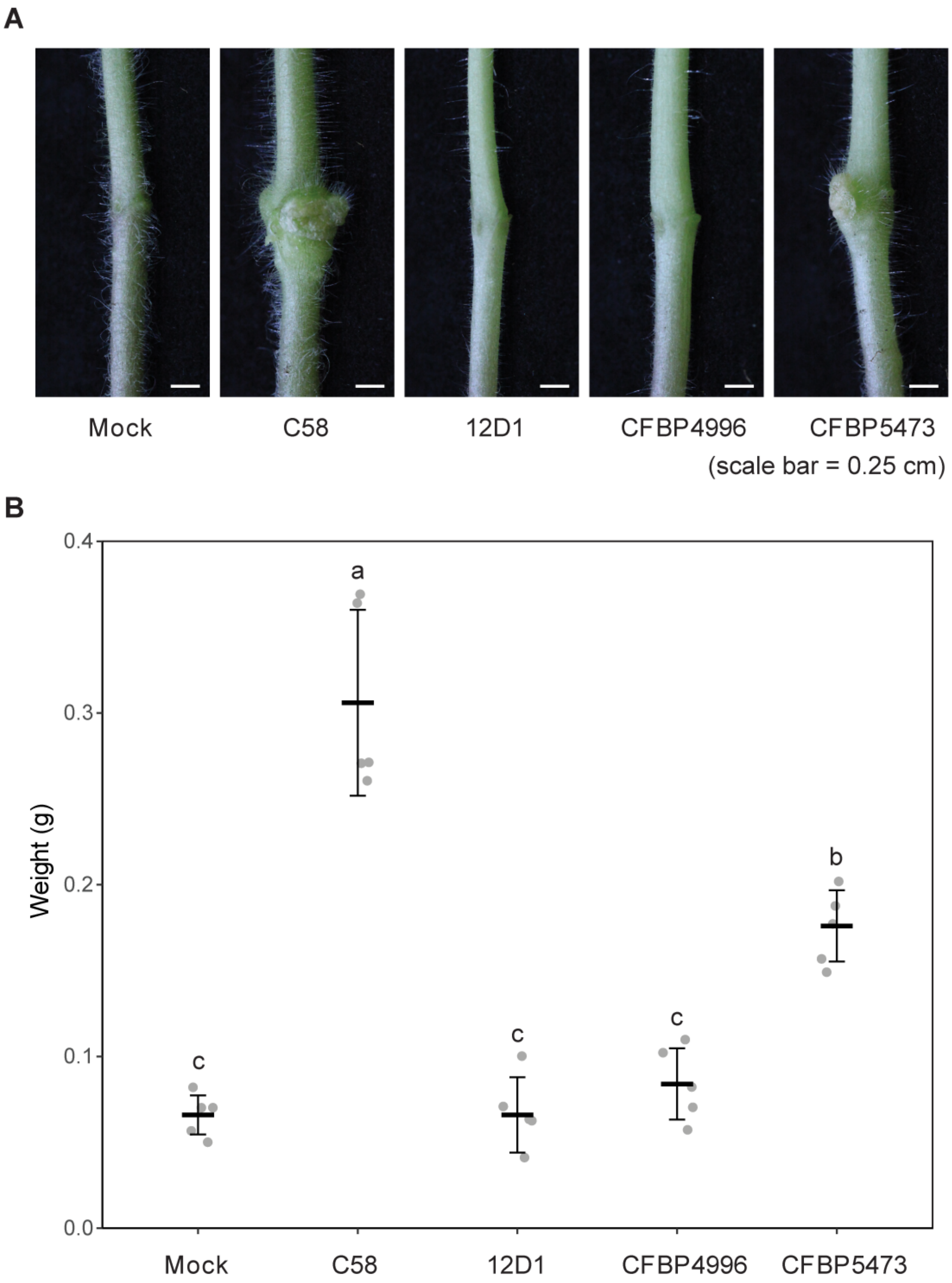

Figure S8. Tomato tumor assay of strains 12D1, CFBP4996, and CFBP5473. Mock was inoculated with sterilized water as a negative control and strain C58 was included as a positive control. Strain 12D1 harbors a plasmid with opine transporter and catabolism genes but lacks *vir* regulon genes and identifiable T-DNA. CFBP4996 and CFBP5473 harbor novel types of putative tumor-inducing plasmids (pTi). (A) Tomato stems at three weeks after inoculation. Scale bar: 0.25 cm. (B) Weight distribution of five biological replicates (1-cm segments of the stem centered at the inoculation site). The letters indicate ANOVA results.

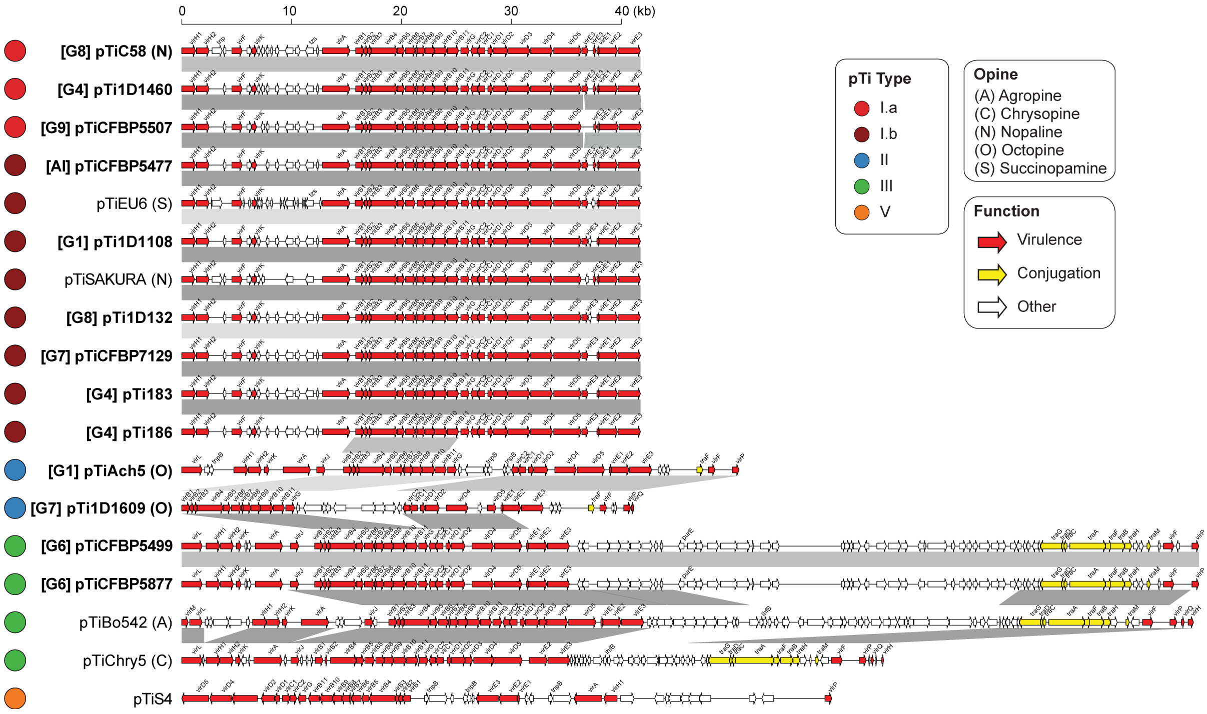

Figure S9. Gene organization of the *vir* regulons on pTi. Syntenic regions are indicated by grey blocks. The virulence (*vir*) genes are highlighted in red, the conjugation (*tra*) genes are highlighted in yellow, and other genes are plotted in white.
